# Translatomics reveals the role of dietary calcium addition in regulating muscle fat deposition in pigs

**DOI:** 10.1038/s41598-024-62986-0

**Published:** 2024-05-29

**Authors:** Jingsu Yu, Xiangling Li, Xinyu Qi, Zhaoxuan Ding, Songtao Su, Lin Yu, Lei Zhou, Yixing Li

**Affiliations:** Guangxi Key Laboratory of Animal Breeding, Disease Control and Prevention, College of Animal Science and Technology, Guangxi University, Nanning, 530004 Guangxi Zhuang Autonomous Region China

**Keywords:** Calcium, Translatomics, Ribo-seq, Intramuscular fat, Pig, Computational biology and bioinformatics, Zoology

## Abstract

Intramuscular fat (IMF) in pork holds significant importance for economic performance within the pig industry and dietary calcium supplementation enhances the accumulation of intramuscular fat. Additionally, calcium ions inhibit translation and reduce protein synthesis. However, the mechanism by which calcium regulates IMF deposition in muscle through translation remains largely unknown. In this study, we compared the ribosome profiles of the longissimus dorsi muscles of Duroc $\times$ Landrace $\times$ Large white pigs from the normal calcium (NC) group or calcium supplement (HC) group by Ribo-seq, and RNA-seq. By integrating multiple-omics analysis, we further discovered 437 genes that were transcriptionally unchanged but translationally altered and these genes were significantly enriched in the oxidative phosphorylation signaling pathway. Furthermore, experimental data showed that inhibiting the expression of COX10 and mtND4L increased triglyceride accumulation in C2C12 cells, providing new targets for intramuscular fat deposition. Finally, this work links dietary calcium, translation regulation and IMF deposition, providing a new strategy for both meat quality and economic performance within the pig industry.

## Introduction

High-quality pork is growing in popularity among customers as people’s living levels rise. The quality of meat is influenced by various factors, with intramuscular fat (IMF) content being the primary factor^1^. Previous studies have exhibited that a high IMF content is believed to promote sensory experience during consumption, as IMF positively influences the juiciness, palatability, and flavor of various types of meat^2,3^. For a prolonged duration, intensive selection for growth rate and lean meat percentage has significantly enhanced the economic advantages of lean pigs but resulted in a significant reduction in meat quality traits^4^. Unfortunately, the simultaneous enhancement of carcass lean percentage and IMF content has become a conundrum. Therefore, it is crucial for animal performance research to explore strategies that can reduce carcass adiposity while regulating the increase in IMF.

Nutritional strategies based on increasing dietary calcium have been demonstrated as a successful approach to pig feeding, giving rise to enhanced IMF accumulation and reduced subcutaneous adiposity^5,6^. Brian Jensen and Pilar Parra et al. revealed that adipose differentiation was impeded by high concentrations of calcium through a receptor-mediated G-protein coupling mechanism^7,8^. Moreover, a diet replete with calcium and vitamin D was found to significantly decrease body weight and fat mass by 26–39% in rats through the downregulation of fatty acid synthase (FAS) gene expression in promotion of lipolysis^9,10^. These results displayed a potential relationship between dietary calcium and carcass adiposity. The excessive supplementation of calcium sources in the diet can increase muscle calcium ion concentration^11^. In addition, targeting supplementation of high levels of vitamin D3 can increase intracellular calcium and activate the calcineurin pathway, thereby improving meat color and pork quality^12^. A previous study has demonstrated that dietary calcium (1.5%) significantly improved meat color and increased intramuscular fat content of longissimus dorsi muscle in pigs^6^. However, the specific mechanism by which calcium promotes the increase of IMF has not been clearly reported.

Translational regulation is a critical step in the process of gene expression, controlling the synthesis of proteins from mRNA and playing a vital role in regulating more than half of gene expression^13^. Calcium ion is of considerable importance in the regulation of eukaryotic protein translation^14^. Since 1990, accumulating evidence has shown that calcium reduces protein synthesis and causes neurite growth arrest by inhibiting the expression of eukaryotic elongation factor 2 (eEF2)^15,16^. In addition, inhibiting the store of intracellular calcium ion reduced protein synthesis and led to the disappearance of multimers in rat acini and rabbit pancreatic lobules^17,18^. These results may be attributed to variations in the regulation of translation by calcium due to differences in cell or tissue-specific characteristics. However, the precise mechanism by which calcium regulates fatty acid metabolism in muscle through protein translation remains poorly understood.

Supplementation with dietary calcium promotes IMF deposition in pigs, and the role of calcium in the regulation of translation has been gradually uncovered. However, the specific role of calcium in the regulation of IMF through translation regulation remains to be further elucidated. In this study, translatomics and transcriptome sequencing were performed on the longissimus dorsi muscle of six intact male pigs (Duroc × Large White × Landrace cross-breed) to explore the translational regulation mechanism of calcium ions on IMF and identify differentially expressed genes (DEGs). Finally, we identified novel functional genes, Both cytochrome c oxidase assembly protein (COX10) and mitochondrial NADH dehydrogenase subunits 4L (mtND4L), whose translation regulated by calcium ion and is involved in IMF accumulation. These findings suggest novel mechanistic approaches for the use of calcium supplementation to enhance meat quality in animals.

## Materials and methods

### Animal ethics statement

All the experimental procedures applied in this study were in accordance with the guidelines of the care and use of laboratory animals of the Guangxi University. Animal experiments were permitted by the Committee on the Ethics of Animal Experiments of Guangxi University (No. GXU2020-287). We report the current study in accordance with ARRIVE guidelines.

### Experimental animals, diets, and design

A total of 16 Duroc × Landrace × Large white pigs, aged 120 days, with an average body weight (BW) of 108.357 ± 5.300 kg, were chosen for the examination. The pigs were bought from Guangxi Jinling Farming Group Co. LTD, Nanning, China. Boar-castrated pigs were divided into two groups by BW: the normal calcium group (NC group, n = 8) and the calcium supplement group (HC group, n = 8). As previously mentioned, calcium carbonate was utilized to modify the diet's calcium content^6^. The HC group received a diet containing 1.5% Ca, while NC group received a diet containing 0.9% Ca^6^.

The pigs in this experiment were raised in a controlled environment with natural ventilation, a temperature of 20 to 25 °C, and unlimited access to water. They also received three meals a day of food. The pigs were sampled after 60 days of feeding. According to the National Standards of the People's Republic of China (GB/T 39235-2020), the diets were designed to meet the nutritional needs of swine (meat-type pigs). Tables 1 and 2 display the composition and nutritional values of the trial diets. Based on the People’s Republic of China’s National Standards of GB/T 6436-2018, the amounts of calcium in the experimental diet were measured^19,20^. After the experiment began, the pigs were weighed on days 0 and 60. These weights were used to calculate the average daily growth rate (ADG; n = 8 per treatment).

**Table 1:** Ingredients of the experimental basal diet (%, as-fed basis).

Ingredients	NC group	HC group
Corn	63.48	62.50
Soybean meal	22.46	22.12
Wheat bran	2.93	2.88
rice skin	4.88	4.80
Pre-mixes^a^	3.91	3.85
Stonewash	2.34	3.85
Total	100.00	100.00

NC, normal calcium diet; HC, calcium supplement diet.

^a^Provided per kilogram of complete diet: 3000 IU vitamin A; 2000 IU vitamin D3; 25 mg vitamin E; 3.5 mg of vitamin K3; 1.0 mg of vitamin B1; 2.0 mg of vitamin B2; 1.5 mg of vitamin B6; 0.010 mg of vitamin B12; 9.5 mg of nicotinic acid; 6.25 mg of calcium pantothenate; 0.25 mg of folic acid; 0.08% lysine; 125 mg of choline chloride; 0.025 mg of biotin; 0.82 mg of Co; 15.63 mg of Cu; 150 mg of Fe; 62.5 mg of Mn; 62.5 mg of Zn; 0.22 mg of Se; 0.75 mg of I.

**Table 2:** Nutrient levels of the experimental basal diet (%, as-dry matter basis).

Nutrient levels	NC group	HC group
Calcium	0.9	1.5
Total phosphorus	1.19	1.2
Crude ash	8.4	8.3
Crude fiber	8.51	8.4
Crude fat	9.3	9.2
Crude protein	44.2	44
Moisture	27.5	27.4

NC,  normal calcium diet; HC,  calcium supplement diet.

### Sample collection and processing

At the end of the experiment, the pigs were euthanized through bloodletting. Samples were taken from the selected pigs’ blood, backfat, and longissimus dorsi muscle. A portion of the muscle samples from the longissimus dorsi were measured for PH value, and Water holding capacity, in accordance with the guidelines provided in the Technical Specification for Determination of Pork Quality (NY/T 821-2019), which was released by the Ministry of Agriculture of the People's Republic of China^21,22^. The procedures described in The Determination of Shear Force for Meat Tenderness (Chinese Standard NY/T 1180-2006) were followed in order to calculate the shear force. The Electrical conductivity was measured according to the method of Driessen et al.^23^. The meat color of the longissimus dorsi muscle was measured using a meat color tester (OPTO-STAR, Matthaus) according to the method of Zhang et al.^6^. The experimental design of each sample was concealed from the testers, while the longissimus dorsi muscle was promptly dissected. The longissimus dorsi muscle samples from the lightest and heaviest pigs in each group were discarded, and the remaining six pigs in each group were collected and randomly paired to form three biological replicates for further sequencing.

### Ribo-seq library construction and sequencing

According to the previous study, with a small adjustment, the ribosome profiling and sequencing were carried out by a commercial service firm (Chi Biotech, Guangzhou, China)^13^. Total Ribosome profiling sequencing (Ribo-seq) libraries from the longissimus dorsi muscle of three biological replicates in Duroc × Landrace × Large white pigs were generated previously. In a nutshell, ribosome protected mRNA is obtained through MNase digestion and RNA purification. Briefly, the longissimus dorsi muscle samples were ground in liquid nitrogen and extracted with 800 μL of polysome lysis buffer (20 mM Tris pH 7.5, 150 mM NaCl, 5 mM MgCl_2_, 24 U/mL TurboDNase, 100 μg/mL cycloheximide, 1 mM dithiothreitol (DTT), 1% Triton X-100, and protease inhibitor mixture) for 30 min. For Ribo-seq, equal amounts of total RNA were present in the sample lysates, which were then digested with 1 U/g RNase I (Epicentre) by shaking the mixture for 30 min at 750 rpm at 24 °C. Nuclease digestion reactions were promptly cooled and spun, and 15 μL of RNase inhibitor was added.

Ribo-seq libraries were prepared according to reference with a minor modification^13^. In total, 5 μg of ribosome-protected mRNA was used to prepare a library with the NEBNext Multiple Small RNA Library Prep Set for Illumina HiSeqX Ten. Briefly, adapters were added to both ends of Ribosome footprints (RFPs), followed by reverse transcription and PCR amplification. Raw reads containing more than 50% low quality bases, or more than 10% N bases were discarded. The FANSe2 algorithm was used to retain, and map reads between the lengths of 15 and 35 to the reference genome, while the edgeR package’s RPKM (Reads Per Kilobase of Transcript per Million mapped Reads) function was used to normalize the relative abundance between two groups. Genes with a fold change ≥ 2 and a *P*-value < 0.05 in a comparison were considered as differentially expressed genes (DEGs) at translational level using R package (http://www.rproject.org/). There were three biological replicates used.

### RNA-seq library construction and sequencing

The total RNA of a longissimus dorsi muscle sample was extracted by TRIzol reagent (Ambion, Inc., Austin, TX, USA) according to the manufacturer’s protocol. The quality of the total RNA was determined by electrophoresis using agarose gel electrophoresis, and the concentration of RNA was measured by NanoDrop 2000 spectrophotometers (Thermo Fisher, MA, U.S.A.). Briefly, the mRNA was purified by Oligo (dT) magnetic beads and separated into short fragments using fragmentation buffer. The resulting cDNA was then end repaired, poly(A) was added, and Illumina sequencing adapters were ligated after being purified using a QiaQuick PCR extraction kit (QIAGEN Company, Dusseldorf, Germany). RNA libraries were prepared according to the VAHTS mRNA-seq v.3 Library Prep Kit for Illumina protocol, and the raw sequencing reads were generated on an Illumina HiSeqX Ten sequencer.

For mRNA sequencing data sets, high quality reads were mapped to a RefSeq mRNA reference sequence (Sus_scrofa.11.1) using the FANSe2 algorithm. Reads that corresponded to different splice variants of the same gene were combined. The mRNA in each sample was normalized using RPKM (reads per kilobase per million reads), whereas the relative abundance between two groups was standardized using the edgeR program. Differentially expressed mRNA were identified using the edgeR program with a fold change > 2 and *P*-value < 0.05. Three biological replicates were used.

### Gene functional annotation

Gene Ontology (GO) and Kyoto Encyclopedia of Genes and Genomes (KEGG) pathway analyses were performed by DAVID. The significance limit for GO term and route identification was set at a *P*-value < 0.05. In order to assess if a preset gene set is significantly enriched or depleted across two phenotypes, we employed a computational method called gene set enrichment analysis (GSEA v2.2.3). By directly extracting pathway names and gene ENTREZ IDs from the KEGG database and translating them into official gene symbols, we created KEGG pathway gene sets files.

### Cell culture and differentiation

C2C12 cells were obtained as previously described^24^. C2C12 cells were cultured in Dulbecco’s Modified Eagle Medium (DMEM) supplemented with 10% fetal bovine serum, 1% penicillin/streptomycin (1.8 mmol/L calcium, Gibco, Beijing, China) in a humidified incubator. The medium’s calcium concentration was adjusted using CaCl_2_. We employed a control group with a calcium concentration of 2 mmol/L and a calcium supplementation group with a calcium concentration of 3 mmol/L. Cells were grown in culture until they were 80% confluent and then differentiated for 7 days in DMEM differentiation media with 2% horse serum (Gibco). After that, cells were exposed to oleic and palmitic acids (OA/PA) for 24 h in order to induce the deposition of fat. DMEM containing oleic acid (200 μM, OA) and palmitic acid (100 μM, PA) was used to mimic the high-fat diet environment in vivo^24^.

### Cell transfection

For the purpose to target COX10 and ND4L, Silencer Select predesigned siRNAs were acquired from a commercial service provider (Sheng gong, GUANZHOU, China). The siCOX10, simtND4L, or negative control siRNA (siNC) were transfected into C2C12 cells to provide an in vitro model of fat accumulation in muscle cells. Before transfection, the media were switched to vehicle DMEM medium (Gibco) with serum free after the cells achieved 60–70% confluence. For transfection, C2C12 myoblasts were plated into a 12-well plate and transfected with plasmids using Hieff TransTM Liposomal Transfection Regent, following the manufacturer’s instructions. Table S1 contains the siRNA sequences.

### Oil red O staining

Longissimus dorsi muscle samples from pigs were cut into frozen sections, soaked in oil red dye solution for 10 min, and then stained with hematoxylin reverse for 2 min. Finally, microscope inspection, image acquisition and analysis were performed.

### Intercellular/tissue triacylglycerol (TG) assay

The intercellular/tissue triglyceride (TG) kit (Nanjing Jiancheng Bioengineering institute, Nanjing, China) was used to determine triglyceride content, following the manufacturer’s instructions. Each well’s cells were filled with 300 μL of RIPA lysis buffer containing 3 μL PMSF, and the cells were then incubated at 4℃ for 30 min. For tissue sample, 50 mg of tissue was placed in a centrifuge tube along with 1 mL of RIPA lysis solution and 10 μL PMSF. The lysate was centrifuged for 10 min at 4 °C and 12,000 rpm to extract the supernatant for triglyceride and protein analysis.

### Quantitative RT-PCR

Total RNA was isolated using TRIzol reagent (GenStar) from C2C12 cells, and then reverse-transcribed with ABScript III RT Master Mix to cDNA following the manufacturer’s instructions. Quantitative real-time PCR analysis was performed, and that cDNA was quantified using 2X Realstar Green Fast Mixture (Takara, Japan) as previously described. To calibrate the relative expression levels, the housekeeping gene β-actin served as the reference gene. Table S2 contains the necessary primer sequences.

### Surface sensing of translation (SUnSET) assay

By detecting the amount of puromycin incorporation, the SUnSET test calculates the rate of mRNA translation^25^. After receiving treatment in calcium supplemented medium for 24 h, C2C12 cells were incubated for 30 min with 10 mg/ml puromycin (Cat#A1113803, Thermo Fisher Scientific). To eliminate any remaining puromycin, cells were washed with PBS. They were then lysed with 1 × RIPA buffer, run on an SDS-PAGE gel, and transferred to a PVDF membrane for reaction with anti-puromycin antibody (Millipore, Cat#MABE343).

### Western blotting

Tissue and cell proteins were extracted with RIPA lysis buffer and 1% protease inhibitor mixture (Solarbio). After undergoing sodium dodecyl sulfate–polyacrylamide gel electrophoresis, protein samples (20 µg) were transferred to PVDF membranes for reaction with antibodies against target genes (Details of the antibodies we used to be listed in Table S3). The membrane was incubated with each antibody for a whole night at 4 °C. After that, they were washed three times by TBST buffer and incubated with corresponding HRP-conjugated secondary antibodies (1:3000) for 1 h at room temperature. The Bio-Rad imaging equipment (Bio-Rad Universal Hood II; Bio-Rad, Hercules, CA, USA) was utilized to image the membrane, and Image J software was employed for quantitative analysis.

### Statistical analysis

All data obtained are expressed as mean ± SD, data analysis was performed with a two-tailed Student’s t-test in two groups. At least three replicates were utilized in each group. GraphPad Prism Version 8.0 software was used for the statistical analysis, and the statistical significance level was set at *P*-value < 0.05.

## Results

### Growth performance and meat quality traits in pigs

Regarding growth performance, there was no discernible difference between the NC and HC groups’ initial BW (*P* > 0.05); however, the HC group’s final BW and ADG were higher (*P* < 0.01) and its backfat thickness was lower (*P* < 0.01) (Table 3). In terms of longissimus dorsi muscle meat quality traits, the HC group exhibited an increase (*P* < 0.05) in meat color and intramuscular TG when compared to the NC group. Nevertheless, there were no significant differences (*P* > 0.05) observed in PH_0h_, PH_24h_, electrical conductivity, water holding capacity, and shear force between the two groups (Table 3).

**Table 3:** Phenotypic record of meat quality traits in pigs.

Item	NC group (n = 8)	HC group (n = 8)	*P*-value
Initial BW (kg)	107.5 ± 4.504	108.75 ± 5.701	*P* > *0.05*
Final BW (kg)	131.875 ± 2.588	141.000 ± 4.986**	*P* < *0.01*
ADG (kg)	0.772 ± 0.044	0.967 ± 0.033**	*P* < *0.01*
Backfat thickness(cm)	2.783 ± 0.194	1.933 ± 0.339**	*P* < 0.01
PH_0h_	5.811 ± 0.237	5.881 ± 0.344	*P* > 0.05
PH_24h_	5.476 ± 0.381	5.296 ± 0.636	*P* > 0.05
Electrical conductivity	1.460 ± 0.342	1.85 ± 0.469	*P* > *0.05*
Water holding capacity (ms)	1.460 ± 0.312	1.92 ± 0.437	*P* > 0.05
Shear force (N)	2.800 ± 0.699	3.282 ± 0.757	*P* > 0.05
Meat color	72.215 ± 7.544	83.353 ± 3.021*	*P* < 0.05
Intramuscular TG (nmol/mg protein)	56.965 ± 5.783	71.062 ± 5.771*	*P* < 0.05

NC,  normal calcium diet; HC,  calcium supplement diet; BW, body weight; ADG,  average daily gain. The asterisk means significant different from Normal calcium diet group, *P < 0.05, **P < 0.01.

### Overview of Ribo-seq and RNA-seq in the Normal calcium and Calcium supplement-diet groups

**Figure 1:**
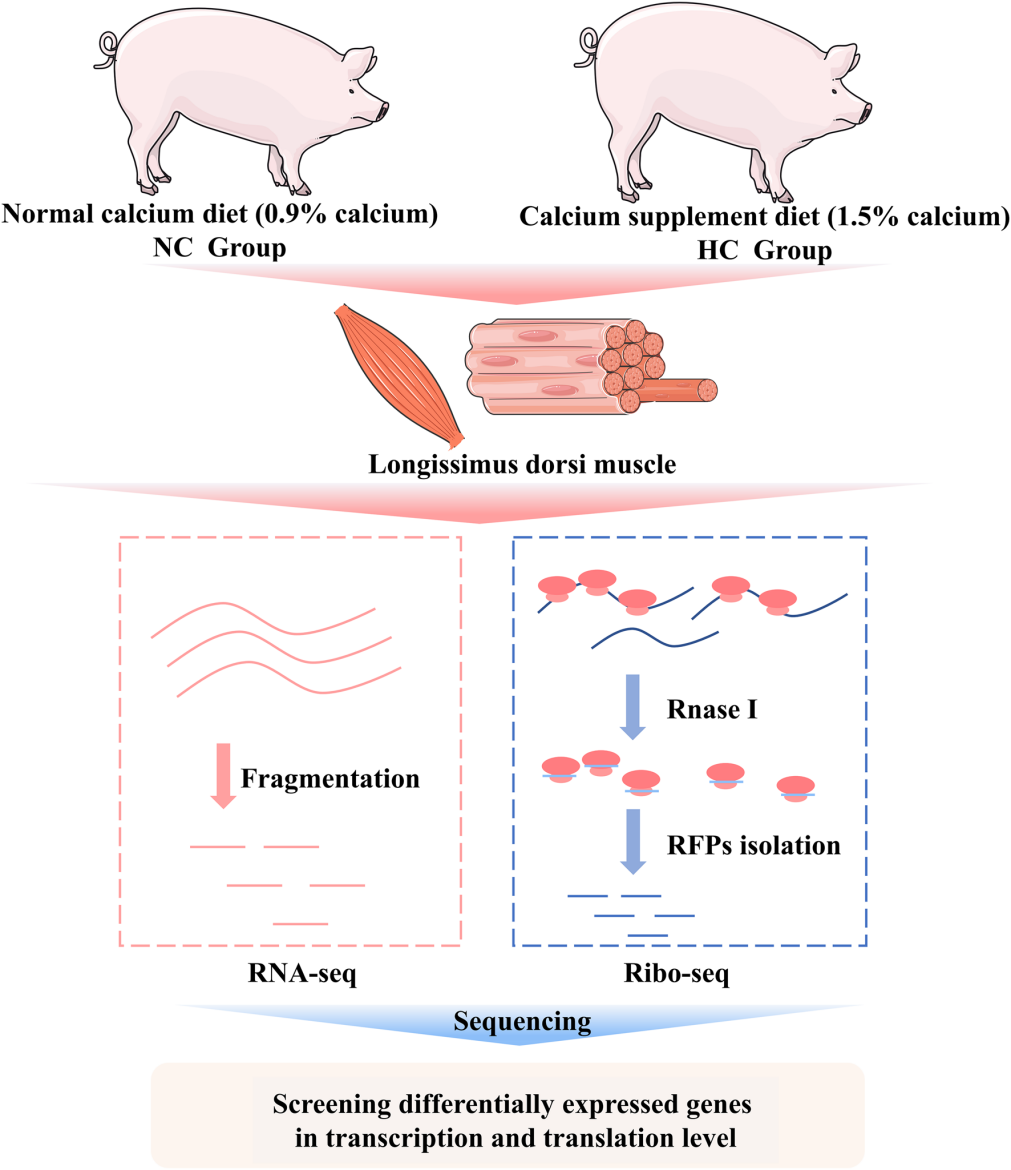
Overview of the experimental design. The longissimus dorsi muscles of the normal calcium (NC) group and the calcium supplement (HC) group were taken for Ribo-seq and RNA-seq detection.

**Figure 2:**
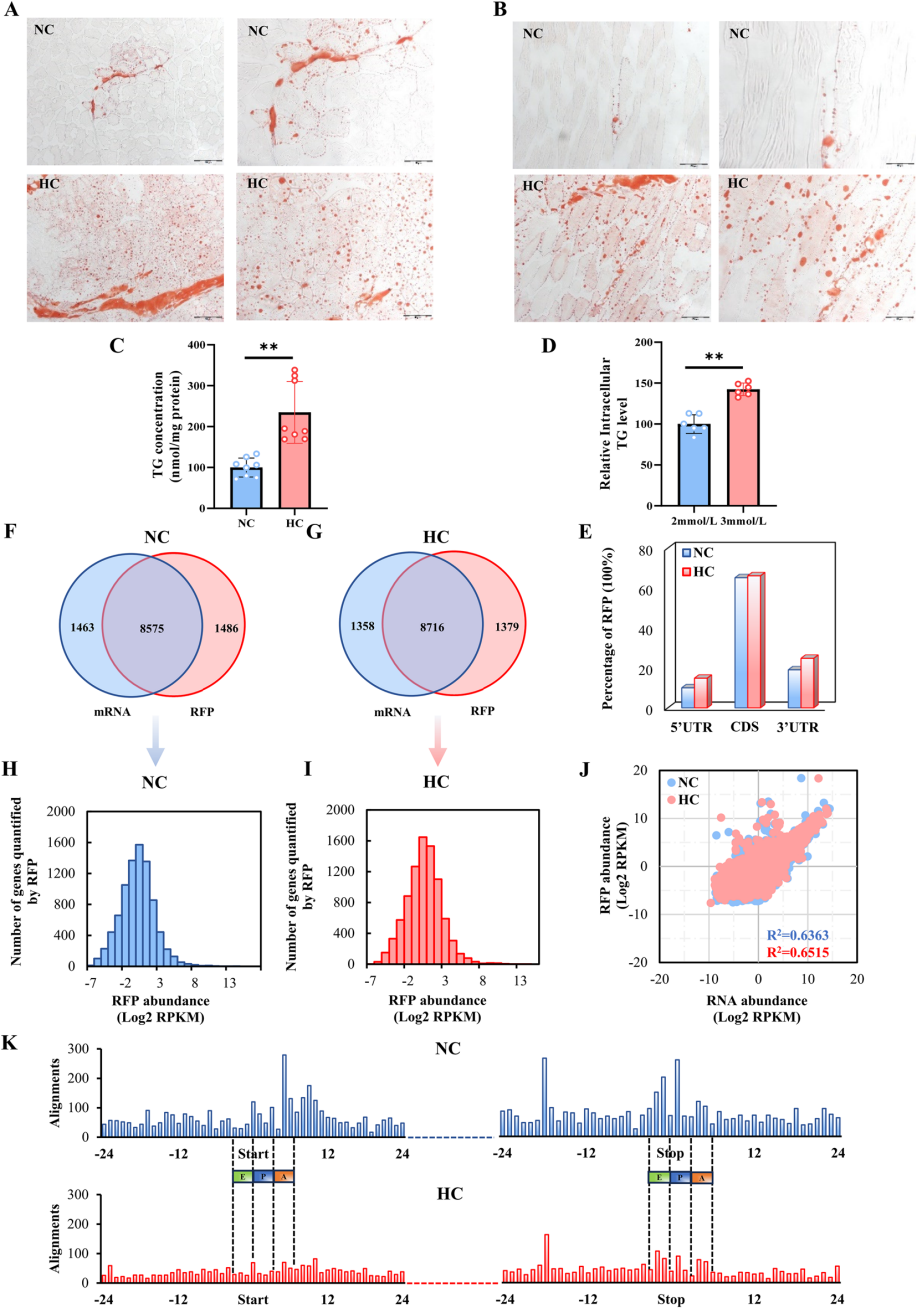
Ribo-seq of the longissimus dorsi muscles in the NC and HC groups. (**A**,**B**) Oil-red O-stained transverse (**A**) and longitudinal (**B**) sections of longissimus dorsi muscle of pigs in the normal calcium (NC) and calcium supplement (HC) groups (n = 8, scale bar: 50 µm or 100 µm). (**C**,**D**) Triglyceride content of muscle tissue (C, n = 8), and C2C12 cells treated with different concentrations of calcium (D, n = 6). (**E**) The percentage of RFPs located in CDS, 5’UTR, and 3’UTR. (**F**,**G**) Venn diagrams of genes identified by RNA-seq and Ribo-seq in the NC and HC groups. (**H**,**I**) Distribution of the RFP abundance. (**J**) Correlation between mRNA and RFP abundance. (**K**) The RFP abundance along CDS start (left) and stop codon regions (right). NC, normal calcium diet; HC, high calcium diet. 2 mmol/L, normal calcium level; 3 mmol/L, calcium supplementation. The data are expressed as the mean ± SD. *P-value < 0.05, **P-value < 0.01.

To understand the molecular mechanism by which calcium promotes intramuscular fat deposition from a translational perspective, we compared the ribosome profiles of the longissimus dorsi muscles of trigram pigs from the normal calcium (NC) group or calcium supplement (HC) group by Ribo-seq, and RNA-seq. Figure 1 shows the experimental strategy of the present study. Oil O staining of the longissimus dorsi muscle samples revealed that calcium addition enhanced intramuscular fat formation (Fig. 2A,B), which was further supported by the triglyceride (TG) content of tissue samples and C2C12 cells (Fig. 2C,D). The NC and HC group’s Ribo-seq and RNA-seq libraries were created and sequenced on HiSeqX platforms, yielding 15.7–27.5 million and 9.6–14.6 million clean reads for ribosome profiling in NC and HC pigs, respectively, as well as 16.7–23.1 million and 2.94–21.0 million clean reads for the NC and HC group’s RNA-seq libraries (Table S4). The principal component analysis (PCA) of Ribo-seq and RNA-seq data showed that the NC samples were clustered and discriminated from HC samples (Fig. S1A,B and S2A,B). These results demonstrate high confidence in the distinction between NC and HC samples at the transcriptional and translational levels, as well as the dependable repeatability of our findings. These findings imply that the sequencing data had extremely good quality.

### Ribo-seq features of the longissimus dorsi muscle

We examined the fundamental ribosome profiles of RFPs between the NC and HC groups in order to investigate whether the ribosome footprint characteristic changes in response to calcium level therapy. According to Fig. S1C, the length distribution of RFPs for NC and HC both significantly peaked at 27–32 nt, comparable to the published RFP length distribution for mice and rats^26^. Figure 2E demonstrated that NC and HC had comparable RFP distribution ratio patterns. The vast majority of RFPs, with an average distribution ratio of more than 60%, were found near the CDS of both NC and HC mRNAs. The RFP distribution was similar to that observed in other mammal species^27^. This demonstrated the high quality of our Ribo-seq library.

Additionally, while comparing the mRNA-seq and Ribo-seq datasets, 8,575 genes were overlapped in NC group, and 8,716 genes were overlapped in the HC group, indicating that the majority of mRNAs entered the translation process in both groups (Fig. 2F,G). The average abundance of RFP in the NC and HC groups was approximately log-normally distributed (Fig. 2H,I). Similar distributions were observed when examining the abundance of mRNAs (Fig. S2C,D). In line with the prior study, both the NC and HC groups’ average RFP abundance displayed strong correlations with the mRNAs (both R^2^ > 0.6, *P*-value < 0.0001, Fig. 2J, and Fig. S2E–H). The three-nucleotide periodicity was clearly observed around the start and stop codon regions of RFPs (Fig. 2K). It’s interesting to note that the number of RFPs was much fewer in the HC group compared to the NC group, indicating that the addition of calcium has limited total horizontal translation. In summary, the overall ribosome binding profiles between NC and HC were comparable, but there were clear changes in the control of translation across the different calcium treatments.

### Differential transcriptional in the NC and HC group

**Figure 3:**
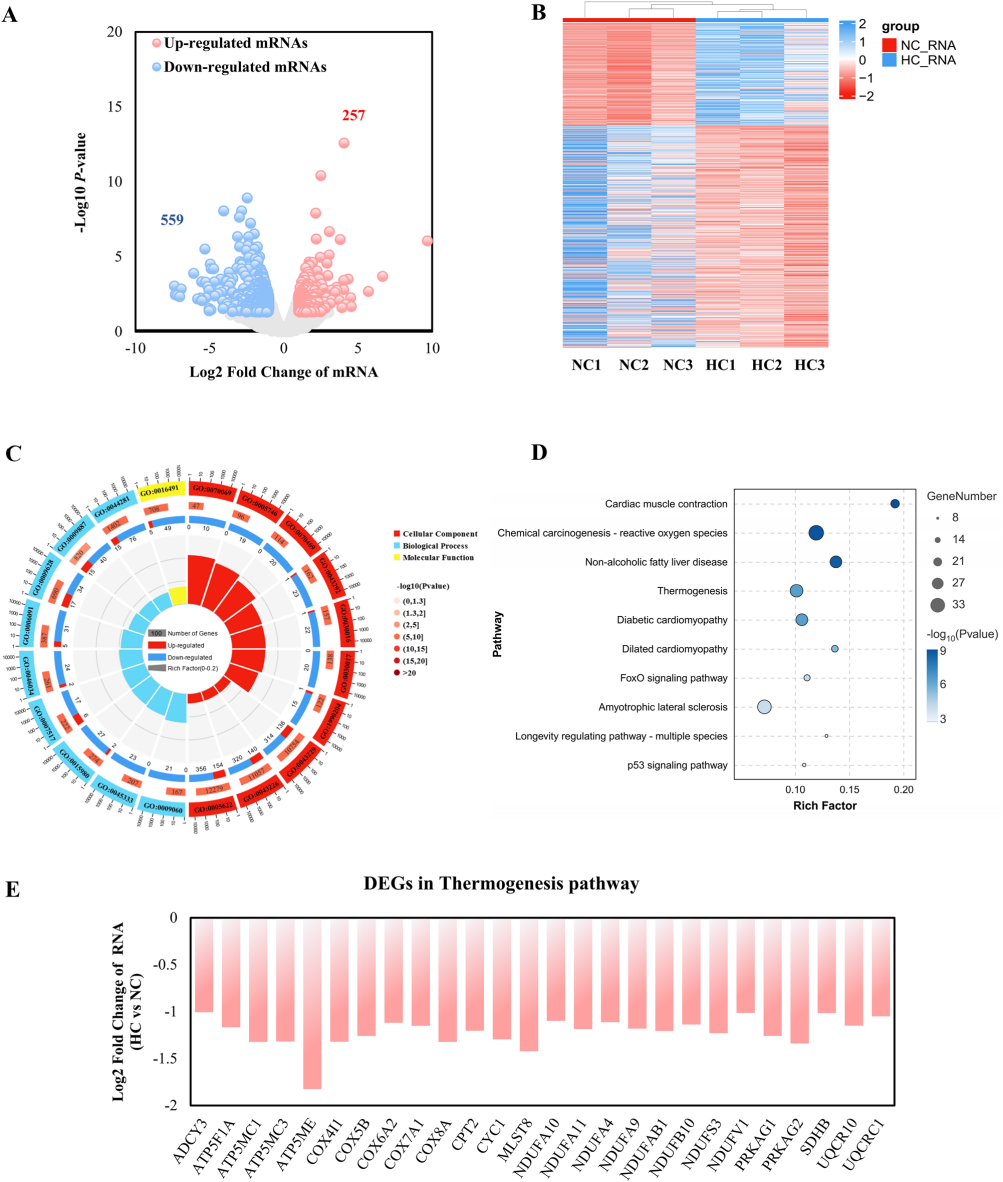
Transcriptome analysis of the NC and HC groups. (**A**) Scatter plot of RNA-seq data. (**B**) Heatmap of the differentially expressed genes in transcription level. (**C**) GO (Gene Ontology) enrichment circle diagram of differentially expressed genes in transcription level (from the outside to the inside, the first circle represents 20 significantly enriched pathways, and the number outside the circle is the coordinate ruler of the number of genes. The second circle represents the number and *P-*value of background genes in this pathway, and the more genes, the longer the bar. The third circle represents the number of DEGs in this pathway (the red color represents upregulated genes and the blue represents downregulated genes). The fourth circle represents the value of Rich Factor in each pathway). (**D**) Bubble chart of Kyoto Encyclopedia of Genes and Genomes (KEGG) pathway enrichment analysis of differentially expressed genes in transcription level. (**E**) Histogram of transcript levels of differentially expressed genes in the thermogenic pathway. X-axis represents 26 selected genes, and Y-axis represents the expression levels of genes from RNA-seq.

Based on RNA-seq data, we were able to examine the transcriptional expression differences between the NC and HC groups. A total of 816 differentially expressed genes (DEGs) were found (Log2 Fold Change ≥ 1 and *P-*value < 0.05), comprising 257 upregulated and 559 downregulated genes (Fig. 3A). The expression patterns of these DEGs are constant within groups but varied across groups, according to the heatmap of these DEGs presented in Fig. 3B. Detailed information for all DEGs is shown in Table S5. In order to investigate the possible role of DEGs in calcium-promoted intramuscular fat deposition, GO term and KEGG pathway enrichment analyses were utilized. According to our research, the biological processes of GO analysis were mostly enriched in cellular respiration (GO:0045333), ATP metabolism (GO:0046034), muscular organ development (GO:0007517), etc., whereas cell components were predominantly enriched in mitochondrial membrane (GO:0005746), respiratory chain complex (GO:0070469), and contractile fiber (GO:0043292) (Fig. 3C). Detailed GO analysis pathways are presented in Table S6. In addition, KEGG analysis revealed that the DEGs were enriched in pathways related to metabolism, including thermogenesis, the FoxO signaling pathway, and the p53 signaling pathway (Fig. 3D). All of the DEGs at the transcription level in the thermogenic pathways were considerably downregulated in the HC group compared to the NC group (Fig. 3E). This shows that decreased muscle thermogenesis and energy metabolism is linked to calcium-promoted muscle fat accumulation.

### Differential translational regulations in the NC and HC groups

**Figure 4:**
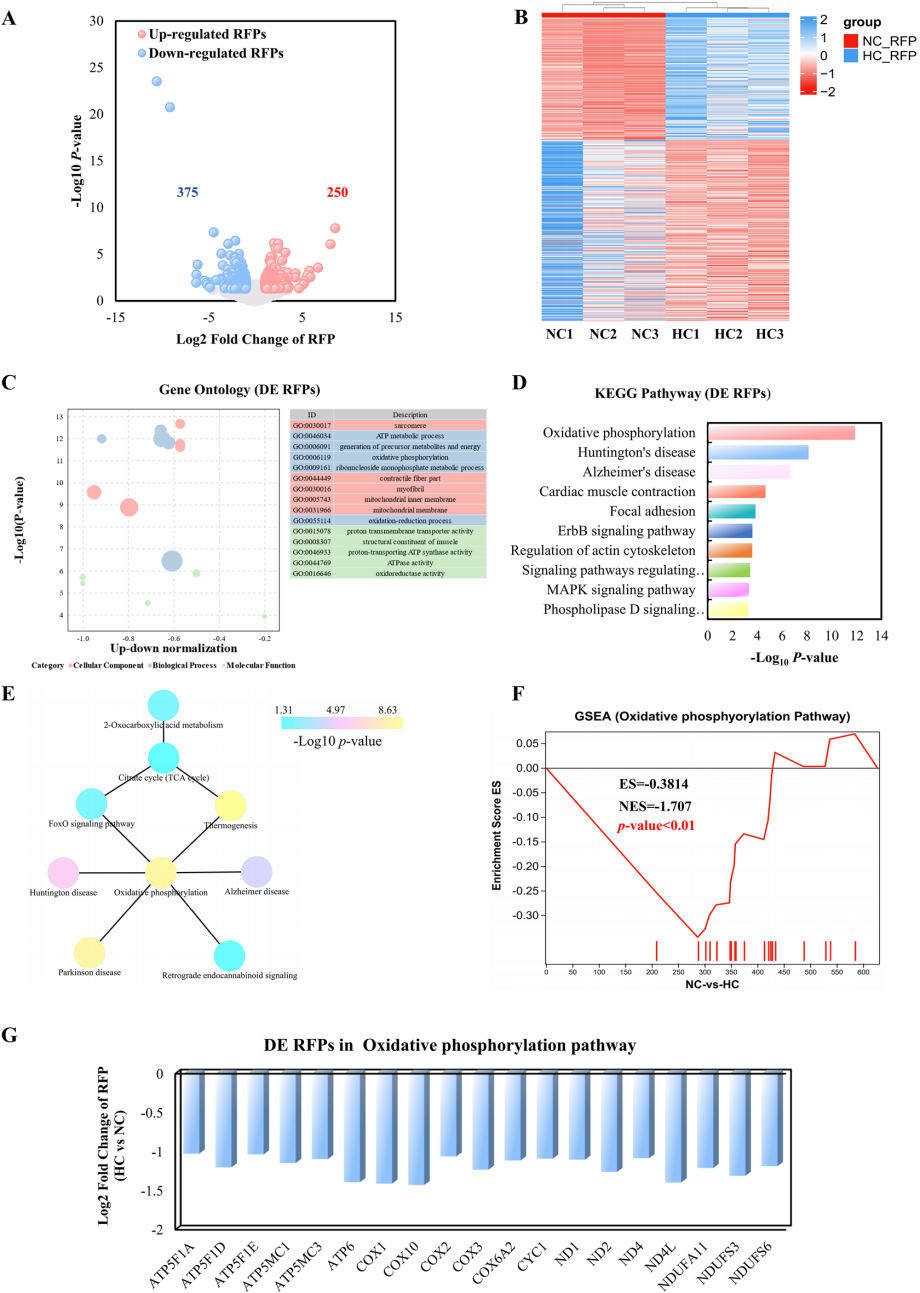
Translational regulation analysis of the NC and HC groups. (**A**) Scatter plot of Ribo-seq data. (**B**) Heatmap of the differentially expressed genes in translation level. (**C**) Bubble chart of GO enrichment analysis of differentially expressed genes in translation level. (**D**) Kyoto Encyclopedia of Genes and Genomes (KEGG) pathway enrichment analysis of differentially expressed genes in translation level. (**E**) PPI analysis of 9 pathway in KEGG using cytoscape. (**F**) GSEA analysis of Ribo-seq data suggested that oxidative phosphorylation signaling pathway was most significantly enriched. (**G**) Histogram of transcript levels of differentially expressed genes in oxidative phosphorylation signaling pathway. X-axis represents 19 selected genes, and Y-axis represents the expression levels of genes from Ribo-seq.

We used a translatomics approach to perform Ribo-seq analysis of the longissimus dorsi muscle, filtering differentially expressed RFPs based on the criteria (Log2 Fold Change ≥ 1 and *P*-value < 0.05), in order to investigate the key functional genes of calcium supplementation on muscle IMF content at the translational level. In summary, 250 upregulated and 375 downregulated genes were found among the 625 differentially expressed RFPs (Fig. 4A). Figure 4B’s heatmap of these differentially expressed RFPs make it clear that there are differences in the expression patterns of DEGs between the two groups. Detailed information for all DEGs is shown in Table S7.

In order to investigate the potential role of DEGs in calcium-promoted intramuscular fat deposition, GO term and KEGG pathway enrichment analysis were used. We found that the biological process of GO analysis was primarily enriched in the ATP metabolic process, generation of precursor metabolism and energy, oxidative phosphorylation, etc., whereas cell components were mainly enriched in proton transmembrane transporter activity, structural constituents of muscle, and ATPase activity (Fig. 4C). Additionally, KEGG analysis revealed that the DEGs were prominent in pathways involved in metabolism, such as the pathways for oxidative phosphorylation, regulation of the actin cytoskeleton, MAPK signaling, and phospholipase D signaling (Fig. 4D). The interaction network of biological processes was also shown using the Cystoscope CLUEGO plug-in (Fig. 4E). The results also showed a considerable enrichment in oxidative phosphorylation (Fig. 4F). All of the translational DEGs in the oxidative phosphorylation pathways were considerably downregulated in the HC group compared to the NC group (Fig. 4G). This suggests that oxidative phosphorylation at the translational level is intimately tied to the stimulation of fat accumulation by calcium.

### Integration of the Ribo-seq results with RNA-seq

To visually reflect the difference between mRNA level and translation level, we compared the differential genes and associated enriched pathways (KEGG pathways or Gene Ontology) between RNA-seq and Ribo-seq in Fig. S3. As shown in Figure S3A, only 12.9% of differentially expressed genes in the transcriptome and translation were overlapped. For the differentially expressed genes in the transcriptome and translation, 33.9% and 35.8% of the enriched pathways overlapped in the KEGG pathway and the GO Biological Process, respectively (Fig. S3B,C). These results suggest differential effects of dietary calcium supplementation on gene expression at the mRNA level and transcript translation.

**Figure 5:**
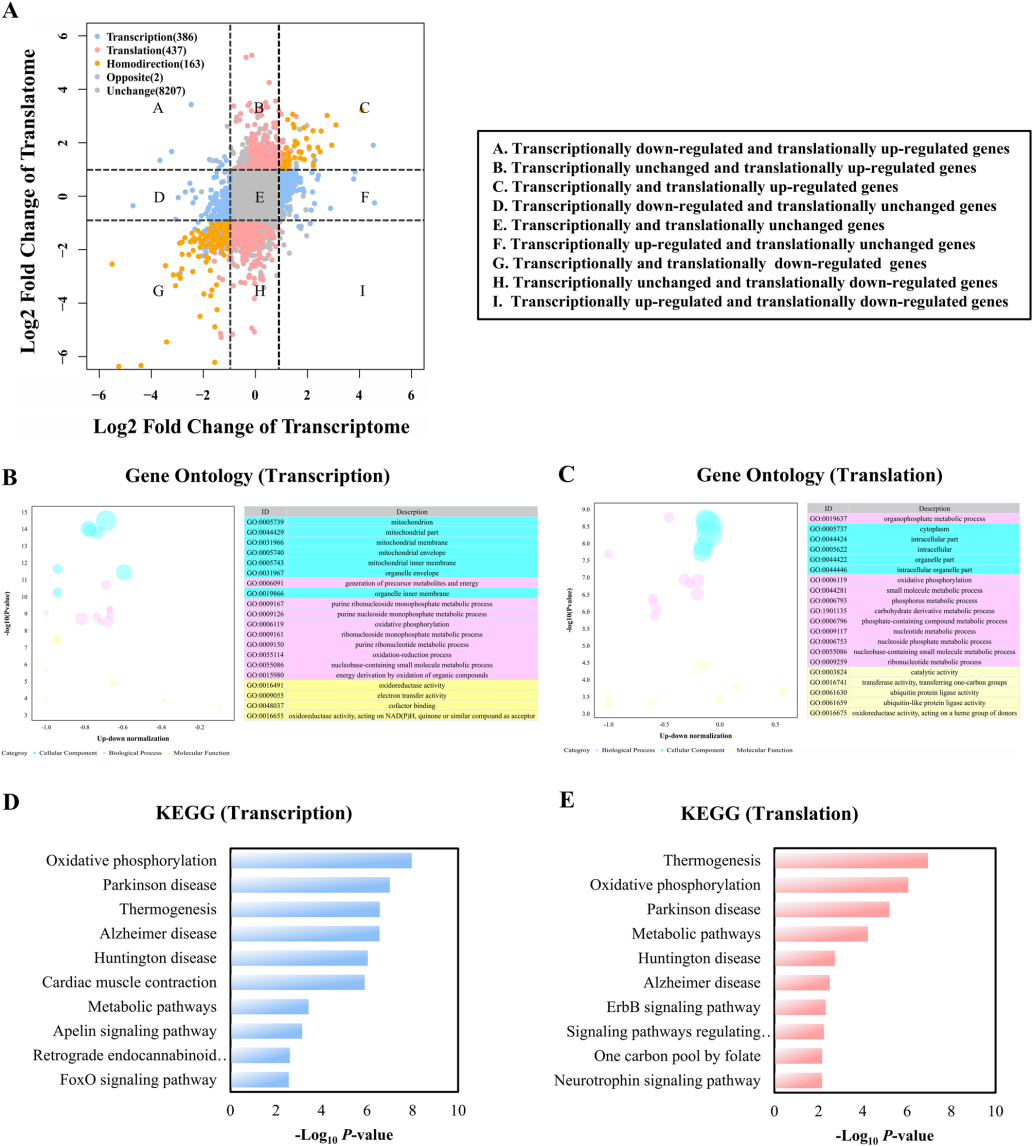
Integration analysis of Ribo-seq and RNA-seq results. (**A**) Scatter plot of the fold change of the HC/NC group at transcriptional and translational levels. (**B**) Bubble chart of GO enrichment analysis of genes in quadrants D, and F. (**C**) Bubble chart of GO enrichment analysis of genes in quadrants B, and H. (**D**) KEGG enrichment analysis of genes in quadrants D, and F. (**E**) KEGG enrichment analysis of genes in quadrants B, and H.

A scatter plot of the fold changes in transcriptional and translational expression is presented in Fig. 5A to clarify the general pattern of changes in gene expression at the transcriptional and translational levels when compared between the HC group and the NC group. According to the criteria, patterns of change were divided into nine groups. The transcriptional and translational regulation of 11.49% of the genes in quadrants B, D, F, and H was inconsistent. GO and KEGG analyses were performed to analyze the gene pathways in which transcripts were translated without difference. Compared to the NC group, genes with no translation difference but differential transcription in the HC group were enriched in precursor metabolites, energy, purine ribonucleoside monophosphate metabolism, and energy pathways, while genes with no transcription difference but differential translation in the HC were enriched in organophosphate metabolism and oxidative phosphorylation pathways (Fig. 5B–E).

**Figure 6:**
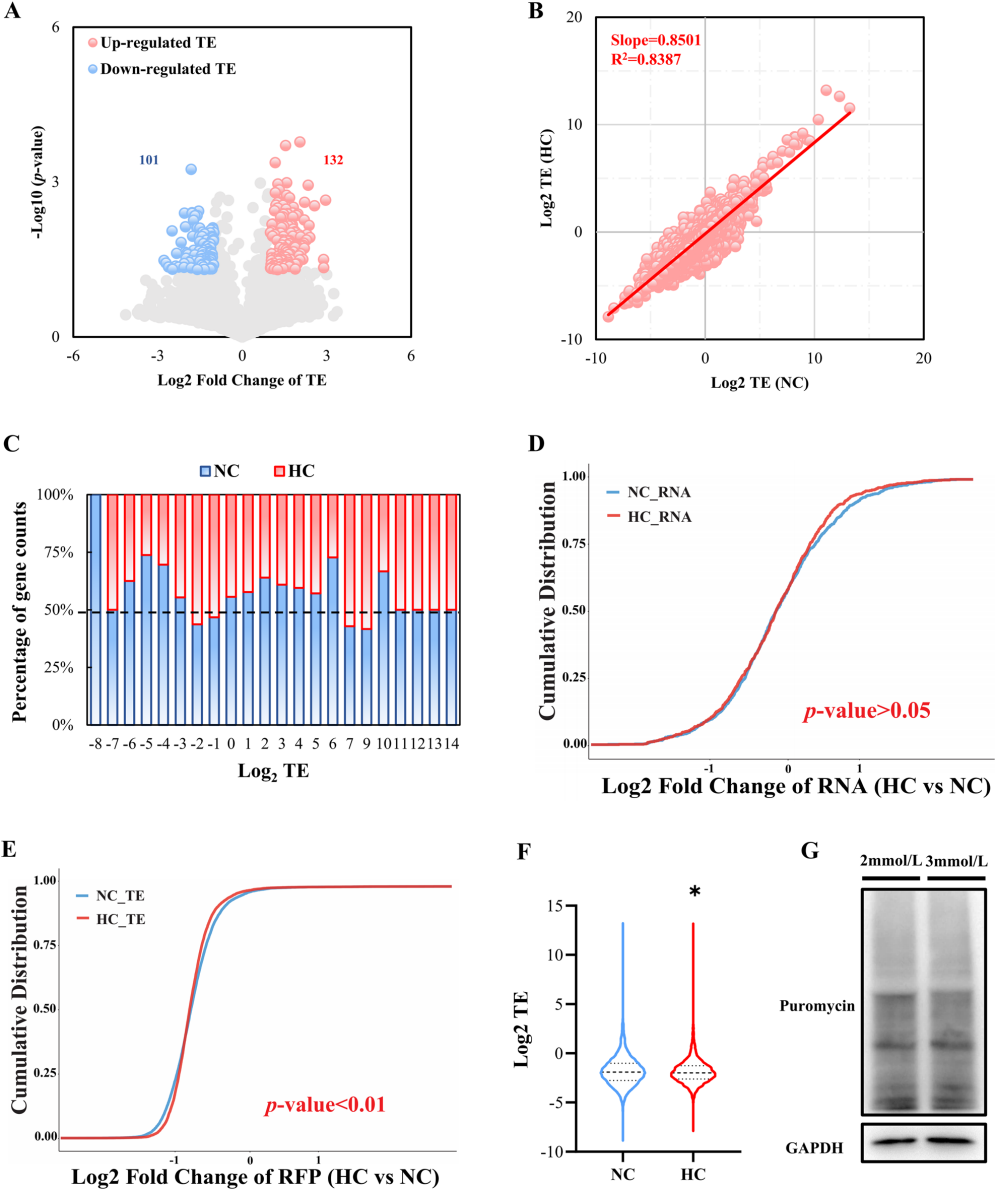
Differences in mRNA translation efficiency between the NC and HC groups. (**A**) Scatter plot of Fold Change of TE. (**B**) Relative TE in the NC and HC groups. (**C**) Relative TE distribution. Genes were classified based on their rounded log2 TE values. For NC or HC groups in each category, every bar represents the percentage of the gene counts in this group to the total gene count. (**D**) The cumulative distribution of genes based on RNA in NC or HC groups. (**E**) The cumulative distribution of genes based on TE in NC or HC groups. (**F**) Violin plot of TE in the NC and HC groups. *P-value < 0.05, Mann–Whitney test. (**G**) Representatives immunoblot of newly synthesized polypeptide labeled by puromycin in C2C12 cells treated with 2 mmol/L and 3 mmol/L CaCl_2_. Gel images were cropped, and the original gels are presented in Supplementary Fig. S5.

Translation efficiency (TE), a crucial translation indicator that measures the efficiency of RNA usage, is calculated as the ratio of the RPKM value of the ribosome map to RNA-Seq^28^. TE values were correlated with transcriptional and translational abundance^29^. A map showing TE’s volcanic alterations may be shown in Fig. 6A. There were 101 genes with considerably lower TE and 132 genes with significantly higher TE. Detailed information for all DEGs is shown in Table S8. A high connection between the average TEs in these two groups was discovered by analyzing the genes found in the pigs of the HC and NC groups (R^2^ = 0.8387, *P*-value < 0.0001; Fig. 6B). The slope, however, was less than 1 (Slope = 0.8501; Fig. 6B). In other words, when the TE value in the NC group was increasing, the TE value in the HC group often grew less than in the NC group. In comparison to the NC group, the HC group had a reduced percentage of genes with higher TEs (Log2 TE ≥ 1). Compared to the NC group, the HC group had a lower percentage of genes with higher TE (Fig. 6C). When examining whether mRNA length or quantity influenced TE, we observed that TE did not correlate well with mRNA abundance, either in the NC or HC groups (Fig. S4A–C). This implies that mRNA length and quantity have minimal effect on how translation is regulated. The ribosome binding mRNA readings in the HC group were considerably lower than those in the NC group (*P*-value < 0.01, Mann–Whitney U test, Fig. 6D,E). The cumulative curves indicated that the HC group’s translation was considerably less than the NC group’s. Additionally, the mean TE of the HC group was lower than that of the NC group (HC mean TE (Log2) = − 1.841, NC mean TE (Log2) =  − 1.789, *P*-value < 0.05; Fig. 6F). Puromycin-treated cells were next submitted to Western blot analysis with whole cell lysates to determine the impact of calcium supplementation on translation in myocytes. Calcium’s inhibitory influence on translation was proven by Sunset (Fig. 6G). These findings collectively imply that calcium supplementation decreases the translation efficiency of transcripts, and that calcium may promote the deposition of IMF by preventing myocyte translation.

### Calcium addition promotes IMF synthesis in pigs by inhibiting gene translation of the oxidative phosphorylation pathway

**Figure 7:**
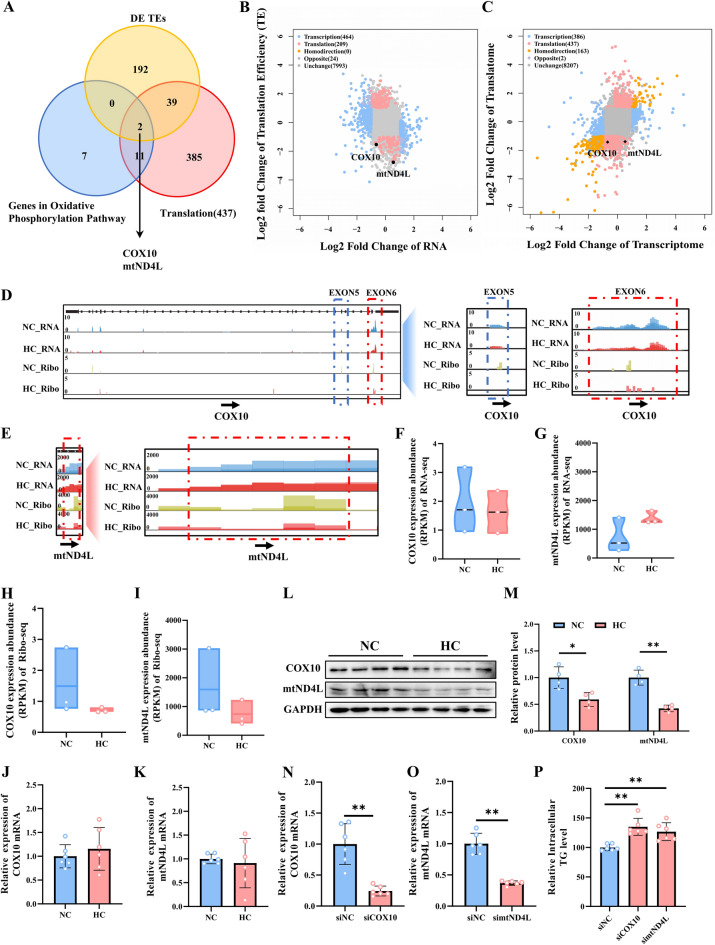
Screening and functional verification of the IMF deposition-related candidate gene. (**A**) Overlap of differentially expressed genes in translation efficiency (TE), oxidative phosphorylation pathway, and translation of nine quadrants. (**B**) Scatterplot showing the translation efficiency (TE) level of COX10 and mtND4L changes at HC compared with NC. (**C**) Scatterplot showing the translational level of COX10 and mtND4L changes at HC compared with NC. (**D**,**E**) IGV snapshot for RNA-seq and Ribo-seq signals of COX10 (**D**) and mtND4L (**E**). (**F**,**G**) Quantification of RNA-seq data for COX10 (F) and mtND4L (**G**). (**H**,**I**) Quantification of Ribo-seq data for COX10 (**H**) and mtND4L (**I**). (**J**,**K**) The mRNA level of COX10 (**J**) and mtND4L (**K**). (**L**) The protein levels of COX10 and mtND4L. Gel images were cropped, and the original gels are presented in Supplementary Fig. S6. (**M**) Quantitative results of COX10 and mtND4L protein levels. (**N**–**O**) The mRNA levels of COX10 (N, n = 6) and mtND4L (O, n = 6) in C2C12 cells after transfected with siNC, siCOX10, and simtND4L. (P) Triglyceride content in C2C12 cells after transfected with siNC, siCOX10, and simtND4L (n = 6). The data are expressed as the mean ± SD. *P-value < 0.05, **P-value < 0.01.

In our investigation, we found that oxidative phosphorylation is related to translational regulation and is essential for HC-induced IMF deposition. Thus, HC-induced intramuscular fat deposition may be influenced by translational regulation. To test this hypothesis and, more crucially, to verify the function of prospective target genes, we screened two genes, COX10 and mtND4L, whose translatome level was substantially linked with oxidative phosphorylation. COX10 and mtND4L genes were screened by the Veen plot (Fig. 7A) and displayed in the nine-quadrant figure (Fig. 7B,C). IGV showed that there was no difference in the transcription level of COX10 and mtND4L in the NC and HC groups (Fig. 7D–G), but the expression levels of them at the translational level in HC were significantly lower than that in the NC group (Fig. 7H,I). Although the mRNA levels of COX10 and mtND4L were the same across the HC and NC groups (Fig. 7J,K), the protein levels of COX10 and mtND4L were lowered in the HC group (Fig. 7L,M). In C2C12 cells, the relative mRNA and protein levels of COX10 and mtND4L were inhibited ~ 50% by siRNA (Fig. 7N,O). In these COX10- and mtND4L-inhibited cells, there was a corresponding 25% rise in intracellular TG levels, respectively. This offers two fresh targets for regulating intramuscular fat (Fig. 7P).

In conclusion, this investigation offers a fresh resource for pinpointing the translational regulation of calcium supplementation involved in controlling IMF deposition in pigs. We discovered numerous possible candidate genes for fat deposition, including COX10 and mtND4L, as well as pathways like oxidative phosphorylation signaling, by combining RNA-seq and Ribo-seq. These prospective candidate genes and pathways are crucial for understanding IMF deposition in pigs, and additional research is needed to determine the precise processes by which they influence IMF content.

## Discussion

IMF content is an important economic trait that directly affects the quality and flavor of pork. The introduction of calcium has been observed to enhance the accumulation of fat within muscle tissue^6^. Earlier research employed transcriptomic analysis to identify messenger RNAs (mRNAs) linked to IMF deposition^4,30^. However, the differentially expressed genes (DEGs) and enrichment pathways identified in these studies exhibited notable variations. Calcium can drive translational changes in a variety of cells and coordinate translation through a variety of mechanisms^16,31–33^. In this study, we have identified potential mRNAs that are influenced by dietary calcium at the translational level, leading to changes in intramuscular fat (IMF) deposition by Ribo-seq combined with RNA-seq analysis.

It is well known that IMF includes extracellular lipids and intracellular lipids. The former is stored in adipocytes and widely distributed in muscle bundles. Muscle progenitor cells are capable of trans-differentiation into non-myogenic cells, including adipocytes, and in addition, adipocytes may also be the adipogenic differentiation of mesenchymal stem cells (MSCs) derived preadipocytes^34,35^. Both mice fed a high-energy diet and obese pigs, especially those with dietary calcium supplementation^6,36^, showed an increase in intramuscular fat content, which is consistent with our study (Fig. 2A–D). Notably, it has been reported that excessive TG accumulation may induce negative feedback regulation of preadipocyte differentiation into mature adipocytes^37^. Consistent with this finding, the significant reduction in backfat thickness induced by dietary calcium supplementation in our study may be an important manifestation of whole-body adipocyte feedback regulation (Table 3). In addition, the feedback regulation of adipocyte differentiation in muscle bundles by high calcium concentrations needs to be further explored. In human disease studies, excessive lipid accumulation in muscle impairs muscle protein synthesis and leads to a decrease in muscle cell proliferation, and also leads to insulin resistance, obesity, and diabetes^38,39^. Therefore, exploring the balanced coordination of myogenesis and lipogenesis in muscle may be important in livestock production.

The inhibition of translational efficiency by calcium ions align with our observation that genes in the HC group exhibit lower TE compared to genes in the NC group^16^. Several investigators have demonstrated that calcium in the endoplasmic reticulum hinders translation initiation in nucleated mammalian cells^40^, and furthermore, elevated calcium levels result in a deceleration of peptide chain elongation^41^. However, several notable disparities have been observed. For instance, Anna S. Hui has documented that calcium plays a crucial role in stimulating the protein synthesis of hypoxia-inducible factor (HIFα) in hypoxic PC12 cells, achieved through the dispersion of HIFα mRNA into the larger multimeric fraction^42,43^. In fact, our findings point to a calcium ion-related regulation pathway (Figs. 4C,D, 5B–E). For more clarification, earlier research has shown that the amount of calcium ions in the cytoplasm affects the contractile state of cardiomyocytes. ^44^. In addition, diabetic cardiomyopathy^45^, amyotrophic lateral sclerosis^46^, Focal adhesion^47^, Alzheimer's disease^48^, Huntington disease^49^, Mitochondrion^50^ and other pathways are intimately associated with the control of calcium ions. In the present investigation, we have successfully showcased in vitro that the existence of calcium ions led to a significant decrease of approximately 20% in the overall translation levels of muscle cells (Fig. 6G). Furthermore, the sequencing results revealed a noteworthy reduction in ribosomal binding RNA reads as a consequence of dietary calcium supplementation (Fig. 6D–F). Hence, it is plausible to hypothesize that the IMF deposition could be attributed to the suppressive impact of calcium ions on translation.

Oxidative phosphorylation plays a crucial role in the process of intramuscular fat accumulation within muscle tissue^43,51,52^. The present investigation revealed that steers exhibited a 45% higher intramuscular fat content compared to regular cattle, accompanied by a notable decrease in the expression of the oxidative phosphorylation protein ATP5B in the longest dorsal length of the steers^53^. The induction of heat shock protein 72 (HSP72) has been found to effectively mitigate triglyceride content in muscle tissue by augmenting the quantity and oxidative capacity of skeletal muscle mitochondria^54,55^. Brain and muscle arnt-like protein 1 (BMAL1) muscle-deficient mice showed increased oxidative capacity and energy expenditure, as well as a further decrease in muscle triglyceride levels^56^. In addition, Silvie Timmers and Xiu-Fang Chen et al. demonstrated that inhibition of oxidative phosphorylation leads to muscle fat storage in rats ^57,58^. Together, these studies highlight that inadequate oxidative phosphorylation is a metabolic pathway that significantly promotes muscle fat accumulation ^59^.

In this study, we identified 625 mRNAs that exhibited differential translation between the HC and NC groups (Fig. 4A). KEGG enrichment analysis revealed a significant enrichment of these differentially translated genes in the oxidative phosphorylation pathway (Fig. 4D). Furthermore, PPI analysis highlighted the oxidative phosphorylation pathway as a central hub pathway (Fig. 4E). Notably, all differentially expressed genes (DEGs) involved in translational regulation within the oxidative phosphorylation pathways were significantly downregulated in the HC group, including ATP5F1A, ATP5F1D, ATP5F1E, ATP5MC1, ATP5MC3, ATP6, COX1, COX2, COX3, COX6A2, COX10, CYC1, mtND1, mtND2, mtND4, mtND4L, NDUFA11, NDUFS3, and NDUFS6 (Fig. 4G). These findings underscore the importance of targeting the oxidative phosphorylation pathway to enhance intramuscular fat content and promote meat quality in animal husbandry.

Mammalian mitochondrial proteins are essential for the proper functioning of the oxidative phosphorylation system^60,61^. Our study demonstrates that siRNA-mediated inhibition of COX10 and mtND4L promotes intramyocellular lipid deposition (Fig. 7N–P). However, the precise role of IMF deposition in this context remains poorly understood. Previous investigations have revealed that COX10 encodes a heme a-farnesyl transferase crucial for assembling the COX with mutations in COX10 being associated with Charcot-Marie-Tooth disease^62^. Interestingly, biochemical and histochemical analyses of liver samples from COX10 knockout mice exhibit severe COX deficiency alongside significant lipid accumulation^63^. In addition, mtND4L is an upstream sequence adjacent to the mtND4L gene on human mtDNA responsible for encoding NADH dehydrogenase subunit 4L involved in various cellular processes such as signal transduction, cancer development, immune response regulation, endocrine function modulation, and nerve signaling^64–66^. For instance, miRNA214 facilitates the progression of chronic kidney disease by disrupting mitochondrial oxidative phosphorylation and suppressing the expression of mtND4L^67^. We hypothesize that these mechanisms contribute to intramuscular fat deposition through a reduction in oxidative phosphorylation and identify COX10 and mtND4L as potential candidate genes for enhancing meat quality in animal husbandry.

## Conclusion

In conclusion, this study presents a novel resource for elucidating the regulatory role of calcium ions in intramuscular fat (IMF) deposition at both transcriptional and translational levels. By integrating Ribo-seq and RNA-seq analyses, we have identified several potential candidate genes associated with fat deposition, such as COX10 and mtND4L, along with their involvement in the oxidative phosphorylation signaling pathway. These candidate genes and pathways demonstrate significant involvement in the regulation of calcium-mediated intramuscular fat (IMF) deposition in pigs, warranting further investigation into their specific mechanisms underlying IMF content modulation. To our knowledge, this is the pioneering study to investigate IMF traits using Ribo-seq combined with RNA-seq sequence analysis. These findings offer valuable insights into comprehending the regulatory mechanisms governing IMF deposition and facilitating genetic enhancement programs for meat quality.

### Supplementary Information

Supplementary Figures.

Supplementary Tables.

## Data Availability

The datasets generated and analyzed during the current study are available from the corresponding author on reasonable request. To review GEO accession GSE264551: Go to https://www.ncbi.nlm.nih.gov/geo/query/acc.cgi?acc=GSE264551

## References

[1] Zhan HW, Xiong YC, Wang ZC, Dong WJ, Zhou QC, Xie SS, Li XY, Zhao SH, Ma YL (2022). Integrative analysis of transcriptomic and metabolomic profiles reveal the complex molecular regulatory network of meat quality in Enshi black pigs. Meat Sci..

[2] Chen D, Wu PX, Wang K, Wang SJ, Ji X, Shen Q, Yu Y, Qiu XT, Xu X, Liu YH, Tang GQ (2022). Combining computer vision score and conventional meat quality traits to estimate the intramuscular fat content using machine learning in pigs. Meat Sci..

[3] Liu YK, Wei YL, Dou YQ, Li CL, Song CL, Zhang Z, Qi KL, Li XJ, Qiao RM, Wang KJ, Li XL, Yang F, Han XL (2023). Effect of miR-149-5p on intramuscular fat deposition in pigs based on metabolomics and transcriptomics. BMC Genom..

[4] Xu Z, Wu JJ, Zhou JW, Zhang Y, Qiao M, Sun H, Li ZP, Li LH, Chen NQ, Oyelami FO, Peng XW, Mei SQ (2022). Integration of ATAC-seq and RNA-seq analysis identifies key genes affecting intramuscular fat content in pigs. Front. Nutr..

[5] Lautrou M, Narcy A, Dourmad JY, Pomar C, Schmidely P, Montminy MPL (2021). Dietary phosphorus and calcium utilization in growing pigs: Requirements and improvements. Front. Vet. Sci..

[6] Zhang ZW, Pan TL, Sun Y, Liu SQ, Song ZY, Zhang HJ, Li YX, Zhou L (2021). Dietary calcium supplementation promotes the accumulation of intramuscular fat. J. Anim. Sci. Biotech..

[7] Jensen B, Farach-Carson MC, Kenaley E, Akanbi KA (2004). High extracellular calcium attenuates adipogenesis in 3T3-L1 preadipocytes. Exp. Cell Res..

[8] Parra P, Bruni G, Palou A, Serra F (2008). Dietary calcium attenuation of body fat gain during high-fat feeding in mice. J. Nutr. Biochem..

[9] He YH (2011). The calcium-sensing receptor affects fat accumulation via effects on antilipolytic pathways in adipose tissue of rats fed low-calcium diets. J. Nutr..

[10] Sun C, Wang L, Yan J, Liu SM (2012). Calcium ameliorates obesity induced by high-fat diet and its potential correlation with p38 MAPK pathway. Mol. Biol. Rep..

[11] Duckett SK, Andrae JG, Pritchard GT, Skow TA, Cuvala SL, Thorngate JH, Sanchez WK (2001). Effects of pre-slaughter administration of oral calcium gel to beef cattle on tenderness. Can. J. Anim. Sci..

[12] Hope-Jones M, Strydom PE, Frylinck L, Webb EC (2012). Effect of dietary beta-agonist treatment, vitamin D supplementation and electrical stimulation of carcasses on colour and drip loss of steaks from feedlot steers. Meat Sci..

[13] Luo ZP, Hu HL, Liu SQ, Zhang ZW, Li YX, Zhou L (2021). Comprehensive analysis of the translatome reveals the relationship between the translational and transcriptional control in high fat diet-induced liver steatosis. RNA Biol..

[14] Brostrom MA, Brostrom CO (2003). Calcium dynamics and endoplasmic reticular function in the regulation of protein synthesis: implications for cell growth and adaptability. Cell Calcium.

[15] Iketani M, Iizuka A, Sengoku K, Kurihara Y, Nakamura F, Sasaki Y, Sato Y, Yamane M, Matsushita M, Nairn AC, Takamatsu K, Goshima Y, Takei K (2013). Regulation of neurite outgrowth mediated by localized phosphorylation of protein translational factor eEF2 in growth cones. Dev. Neurobiol..

[16] Iizuka A, Sengoku K, Iketani M, Nakamura F, Sato Y, Matsushita M, Nairn AC, Takamatsu K, Goshima Y, Takei K (2007). Calcium-induced synergistic inhibition of a translational factor eEF2 in nerve growth cones. Biochem. Biophys. Res. Commun..

[17] Perkins PS, Park JH, Pandol SJ (1997). The role of calcium in the regulation of protein synthesis in the exocrine pancreas. Pancreas.

[18] Wang Q, Heimberg H, Pipeleers D, Ling Z (2008). Glibenclamide activates translation in rat pancreatic beta cells through calcium-dependent mTOR, PKA and MEK signalling pathways. Diabetologia.

[19] Xin H, Wang M, Xia Z, Yu B, He J, Yu J, Mao X, Huang Z, Luo Y, Luo J, Yan H, Wang H, Wang Q, Zheng P, Chen D (2021). Fermented diet liquid feeding improves growth performance and intestinal function of pigs. Animals.

[20] Ma Z, Wang C, Wang B, Yao L, Kong B, Shan A, Li J, Meng Q (2023). Effects of feeding corn distillers dried grains with solubles on muscle quality traits and lipidomics profiling of finishing pigs. Animals.

[21] Menci R, Luciano G, Natalello A, Priolo A, Mangano F, Biondi L, Bella M, Scerra M, Lanza M (2024). Performance and meat quality in pigs fed hydrolysable tannins from Tara spinosa. Meat Sci..

[22] Zhang Q, Cho S, Song J, Jeong J, Yu M, Mun S, Han K, Kim IH (2023). Multi-enzyme supplementation to diets containing 2 protein levels affects intramuscular fat content in muscle and modulates cecal microflora without affecting the growth performance of finishing pigs. Probiot. Antimicrob. Proteins.

[23] Driessen B, Van Beirendonck S, Buyse J (2020). Effects of housing, short distance transport and lairage on meat quality of finisher pigs. Animals.

[24] Liu SQ, Yang D, Yu L, Aluo ZE, Zhang ZW, Qi YL, Li YX, Song ZY, Xu GX, Zhou L (2021). Effects of lycopene on skeletal muscle-fiber type and high-fat diet-induced oxidative stress. J. Nutr. Biochem..

[25] Zhuang YR, Li ZY, Xiong SY, Sun CJ, Li BY, Wu SA, Lyu J, Shi X, Yang L, Chen YT, Bao ZB, Li X, Sun CHW, Chen YL, Deng HT, Li TT, Wu QF, Qi L, Huang Y, Yang XR, Lin Y (2023). Circadian clocks are modulated by compartmentalized oscillating translation. Cell.

[26] Shen ZJ, Zeng LX, Zhang ZH (2020). Translatome and transcriptome profiling of hypoxic-induced rat cardiomyocytes. Mol. Ther.-Nucl. Acids.

[27] van Heesch S, Witte F, Schneider-Lunitz V, Schulz JF, Adami E, Faber AB, Kirchner M, Maatz H, Blachut S, Sandmann CL, Kanda M, Worth CL, Schafer S, Calviello L, Merriott R, Patone G, Hummel O, Wyler E, Obermayer B, Mücke MB, Lindberg EL, Trnka F, Memczak S, Schilling M, Felkin LE, Barton PJR, Quaife NM, Vanezis K, Diecke S, Mukai M, Mah N, Oh SJ, Kurtz A, Schramm C, Schwinge D, Sebode M, Harakalova M, Asselbergs FW, Vink A, de Weger RA, Viswanathan S, Widjaja AA, Gärtner-Rommel A, Milting H, dos Remedios C, Knosalla C, Mertins P, Landthaler M, Vingron M, Linke WA, Seidman JG, Seidman CE, Rajewsky N, Ohler U, Cook SA, Hubner N (2019). The translational landscape of the human heart. Cell.

[28] Huang, Y., Ma, J.Y., Yang, C.Y., Wei, P.J., Yang, M.H., Han, H., Chen, H.D., Yue, T.F., Xiao, S., Chen, X.Y., Li, Z.Q., Tang, Y.L., Luo, J.S., Lin, S.B., & Huang, L.B. (2022) METTL1 promotes neuroblastoma development through m6G tRNA modification and selective oncogenic gene translation. Biomark. Res. **10**. 10.1186/s40364-022-00414-z PMC9454133 36071474

[29] Wu LY, Lv YQ, Ye Y, Liang YR, Ye JH (2020). Transcriptomic and translatomic analyses reveal insights into the developmental regulation of secondary metabolism in the young shoots of tea plants (*Camellia sinensis* L.). J. Agric. Food Chem..

[30] Khan R, Raza SHA, Junjvlieke Z, Wang HB, Cheng G, Smith SB, Jiang ZL, Li AN, Zan LS (2020). RNA-seq reveal role of bovine in the regulation of adipogenesis. Arch. Biochem. Biophys..

[31] Pirouz M, Wang CH, Liu Q, Ebrahimi AG, Shamsi F, Tseng YH, Gregory RI (2020). The Perlman syndrome DIS3L2 exoribonuclease safeguards endoplasmic reticulum-targeted mRNA translation and calcium ion homeostasis. Nat. Commun..

[32] Nairn AC, Matsushita M, Nastiuk K, Horiuchi A, Mitsui K, Shimizu Y, Palfrey HC (2001). Elongation factor-2 phosphorylation and the regulation of protein synthesis by calcium. Progress Mol. Subcellular Biol..

[33] Aktas H, Flückiger R, Acosta JA, Savage JM, Palakurthi SS, Halperin JA (1998). Depletion of intracellular Ca stores, phosphorylation of eIF2α, and sustained inhibition of translation initiation mediate the anticancer effects of clotrimazole. Proc. Natl. Acad. Sci. U.S.A..

[34] Qi RL, Qiu XY, Zhang Y, Wang J, Wang Q, Wu M, Huang JX, Yang FY (2019). Comparison of LncRNA expression profiles during myogenic differentiation and adipogenic transdifferentiation of myoblasts. Int. J. Mol. Sci..

[35] Qiu K, Xu DD, Wang LQ, Zhang X, Jiao N, Gong L, Yin JD (2020). Association analysis of single-cell RNA sequencing and proteomics reveals a vital role of ca signaling in the determination of skeletal muscle development potential. Cells-Basel.

[36] von Rosenberg SJ, Weber GM, Erhardt A, Höller U, Wehr UA, Rambeck WA (2016). Tolerance evaluation of overdosed dietary levels of 25-hydroxyvitamin D in growing piglets. J. Anim. Physiol. Nutr..

[37] Eberlé D, Hegarty B, Bossard P, Ferré P, Foufelle F (2004). SREBP transcription factors: Master regulators of lipid homeostasis. Biochimie.

[38] Robles PG, Sussman MS, Naraghi A, Brooks D, Goldstein RS, White LM, Mathur S (2015). Intramuscular fat infiltration contributes to impaired muscle function in COPD. Med. Sci. Sport Exerc..

[39] Tachi Y, Kozuka A, Hirai T, Kojima Y, Ishizu Y, Honda T, Kuzuya T, Hayashi K, Ishigami M, Goto H (2018). Skeletal muscle fat deposition is associated with hepatocellular carcinoma development in patients with chronic liver disease. Nutrition.

[40] Brostrom CO, Chin KV, Wong WL, Cade C, Brostrom MA (1989). Inhibition of translational initiation in eukaryotic cells by calcium ionophore. J. Biol. Chem..

[41] Laitusis AL, Brostrom CO, Ryazanov AG, Brostrom MA (1998). An examination of the role of increased cytosolic free Ca2+ concentrations in the inhibition of mRNA translation. Arch. Biochem. Biophys..

[42] Hui AS, Bauer AL, Striet JB, Schnell PO, Czyzyk-Krzeska MF (2006). Calcium signaling stimulates translation of HIF-α during hypoxia. FASEB J..

[43] Li R, Wang Y, Yang Z, He Y, Zhao T, Fan M, Wang X, Zhu L, Wang X (2015). Hypoxia-inducible factor-1α regulates the expression of L-type voltage-dependent Ca(2+) channels in PC12 cells under hypoxia. Cell Stress Chaperones.

[44] Williams AJ (1997). The functions of two species of calcium channel in cardiac muscle excitation-contraction coupling. Eur. Heart J..

[45] Kain V, Kumar S, Sitasawad SL (2011). Azelnidipine prevents cardiac dysfunction in streptozotocin-diabetic rats by reducing intracellular calcium accumulation, oxidative stress and apoptosis. Cardiovasc. Diabetol..

[46] Muhlig AK, Steingrover J, Heidelbach HS, Wingerath M, Sachs W, Hermans-Borgmeyer I, Meyer-Schwesinger C, Choi HY, Lim BJ, Patry C, Hoffmann GF, Endlich N, Bracke K, Weiss M, Guse AH, Lasse M, Rinschen MM, Braun F, Huber TB, Puelles VG, Schmitt CP, Oh J (2022). The calcium-sensing receptor stabilizes podocyte function in proteinuric humans and mice. Kidney Int..

[47] Yao M, Tijore A, Cheng D, Li JV, Hariharan A, Martinac B, Tran Van Nhieu G, Cox CD, Sheetz M (2022). Force- and cell state-dependent recruitment of Piezo1 drives focal adhesion dynamics and calcium entry. Sci. Adv..

[48] Wang L, Yin YL, Liu XZ, Shen P, Zheng YG, Lan XR, Lu CB, Wang JZ (2020). Current understanding of metal ions in the pathogenesis of Alzheimer's disease. Transl. Neurodegener..

[49] Khakh BS, Beaumont V, Cachope R, Munoz-Sanjuan I, Goldman SA, Grantyn R (2017). Unravelling and exploiting astrocyte dysfunction in Huntington's disease. Trends Neurosci..

[50] Kirichok Y, Krapivinsky G, Clapham DE (2004). The mitochondrial calcium uniporter is a highly selective ion channel. Nature.

[51] Rico-Sanz J, Hajnal JV, Thomas EL, Mierisová S, Ala-Korpela M, Bell JD (1998). Intracellular and extracellular skeletal muscle triglyceride metabolism during alternating intensity exercise in humans. J. Physiol..

[52] Watt MJ, Cheng YS (2017). Triglyceride metabolism in exercising muscle. BBA-Mol. Cell Biol. Lipids.

[53] Jeong J, Bong J, Kim GD, Joo ST, Lee HJ, Baik M (2013). Transcriptome changes favoring intramuscular fat deposition in the longissimus muscle following castration of bulls. J. Anim. Sci..

[54] Henstridge DC, Bruce CR, Drew BG, Tory K, Kolonics A, Estevez E, Chung J, Watson N, Gardner T, Lee-Young RS, Connor T, Watt MJ, Carpenter K, Hargreaves M, McGee SL, Hevener AL, Febbraio MA (2014). Activating HSP72 in rodent skeletal muscle increases mitochondrial number and oxidative capacity and decreases insulin resistance. Diabetes.

[55] Tsuzuki T, Kobayashi H, Yoshihara T, Kakigi R, Ichinoseki-Sekine N, Naito H (2017). Attenuation of exercise-induced heat shock protein 72 expression blunts improvements in whole-body insulin resistance in rats with type 2 diabetes. Cell Stress Chaperones.

[56] Wada T, Ichihashi Y, Suzuki E, Kosuge Y, Ishige K, Uchiyama T, Makishima M, Nakao R, Oishi K, Shimba S (2018). Deletion of prevents diet-induced ectopic fat accumulation by controlling oxidative capacity in the skeletal muscle. Int. J. Mol. Sci..

[57] Chen XF, Wang L, Wu YZ, Song SY, Min HY, Yang Y, He X, Liang Q, Yi L, Wang Y, Gao Q (2018). Effect of puerarin in promoting fatty acid oxidation by increasing mitochondrial oxidative capacity and biogenesis in skeletal muscle in diabetic rats. Nutr. Diabetes.

[58] Timmers S, Nabben M, Bosma M, van Bree B, Lenaers E, van Beurden D, Schaart G, Westerterp-Plantenga MS, Langhans W, Hesselink MKC, Schrauwen-Hinderling VB, Schrauwen P (2012). Augmenting muscle diacylglycerol and triacylglycerol content by blocking fatty acid oxidation does not impede insulin sensitivity. Proc. Natl. Acad. Sci. U.S.A..

[59] Núñez Y, Radovic C, Savic R, García-Casco JM, Candek-Potokar M, Benítez R, Radojkovic D, Lukic M, Gogic M, Muñoz M, Fontanesi L, Ovilo C (2021). Muscle transcriptome analysis reveals molecular pathways related to oxidative phosphorylation, antioxidant defense, fatness and growth in mangalitsa and Moravka pigs. Animals.

[60] Li T, Han JB, Jia LJ, Hu X, Chen LQ, Wang YG (2019). PKM2 coordinates glycolysis with mitochondrial fusion and oxidative phosphorylation. Protein Cell.

[61] Gustafsson CM, Falkenberg M, Larsson NG (2016). Maintenance and expression of mammalian mitochondrial DNA. Annu. Rev. Biochem..

[62] Diaz F, Thomas CK, Garcia S, Hernandez D, Moraes CT (2005). Mice lacking in skeletal muscle recapitulate the phenotype of progressive mitochondrial myopathies associated with cytochrome oxidase deficiency. Hum. Mol. Genet..

[63] Diaz F, Garcia S, Hernandez D, Regev A, Rebelo A, Oca-Cossio J, Moraes CT (2008). Pathophysiology and fate of hepatocytes in a mouse model of mitochondrial hepatopathies. Gut.

[64] Järviaho T, Hurme-Niiranen A, Soini HK, Niinimäki R, Möttönen M, Savolainen ER, Hinttala R, Harila-Saari A, Uusimaa J (2018). Novel non-neutral mitochondrial DNA mutations found in childhood acute lymphoblastic Leukemia. Clin. Genet..

[65] Xu BY, Zhou ZY, Wen YT, Li ZW, Huang ZX, Li YH (2022). The immunometabolic landscape of the bone marrow microenvironment in acute myeloid leukemia. Exp. Hematol. Oncol..

[66] Xia M, Ruan ZY, Chen B, Wang YQ, Zhou ZZ, Ren SD, Wu L, Tang N (2020). Wuzang Wenyang Huayu decoction regulates differentially expressed transcripts in the rats' hippocampus after cerebral hypoperfusion. J. Cell Mol. Med..

[67] Bai M, Chen H, Ding D, Song R, Lin J, Zhang Y, Guo Y, Chen S, Ding G, Zhang Y, Jia Z, Huang S, He JC, Yang L, Zhang A (2019). MicroRNA-214 promotes chronic kidney disease by disrupting mitochondrial oxidative phosphorylation. Kidney Int..

