# Supplementary material for: Translatomics reveals the role of dietary calcium addition in regulating muscle fat deposition in pigs
Source: Sci Rep. 2024 May 29;14:12295. doi: 10.1038/s41598-024-62986-0 (PMC11136974; doi:10.1038/s41598-024-62986-0)
Supplement: Supplementary file 1 — Supplementary Figures. [file 41598_2024_62986_MOESM1_ESM.pdf]

Translatomics reveals the role of dietary calcium addition in regulating muscle fat deposition in pigs

Authors: Jingsu Yu[1], Xiangling Li[1], Xinyu Qi, Zhaoxuan Ding, Songtao Su, Lin Yu, Lei Zhou*, Yixing Li*,

Affiliation: Guangxi Key Laboratory of Animal Breeding, Disease Control and Prevention, College of Animal Science and Technology, Guangxi University, Nanning, 530004, China.

* Corresponding authors:

Prof. Lei Zhou, College of Animal Science and Technology, Guangxi University, Nanning city, Guangxi Zhuang Autonomous Region, China, 530004. Tel.: 0086-0771-3235635, E-mail: zhoulei@gxu.edu.cn

Prof. Yixing Li, College of Animal Science and Technology, Guangxi University, Nanning city, Guangxi Zhuang Autonomous Region, China, 530004. Tel.: 0086-0771-3231158, E-mail: liyixing39@gxu.edu.cn

[1]These authors contributed equally to this work.

Figure S1

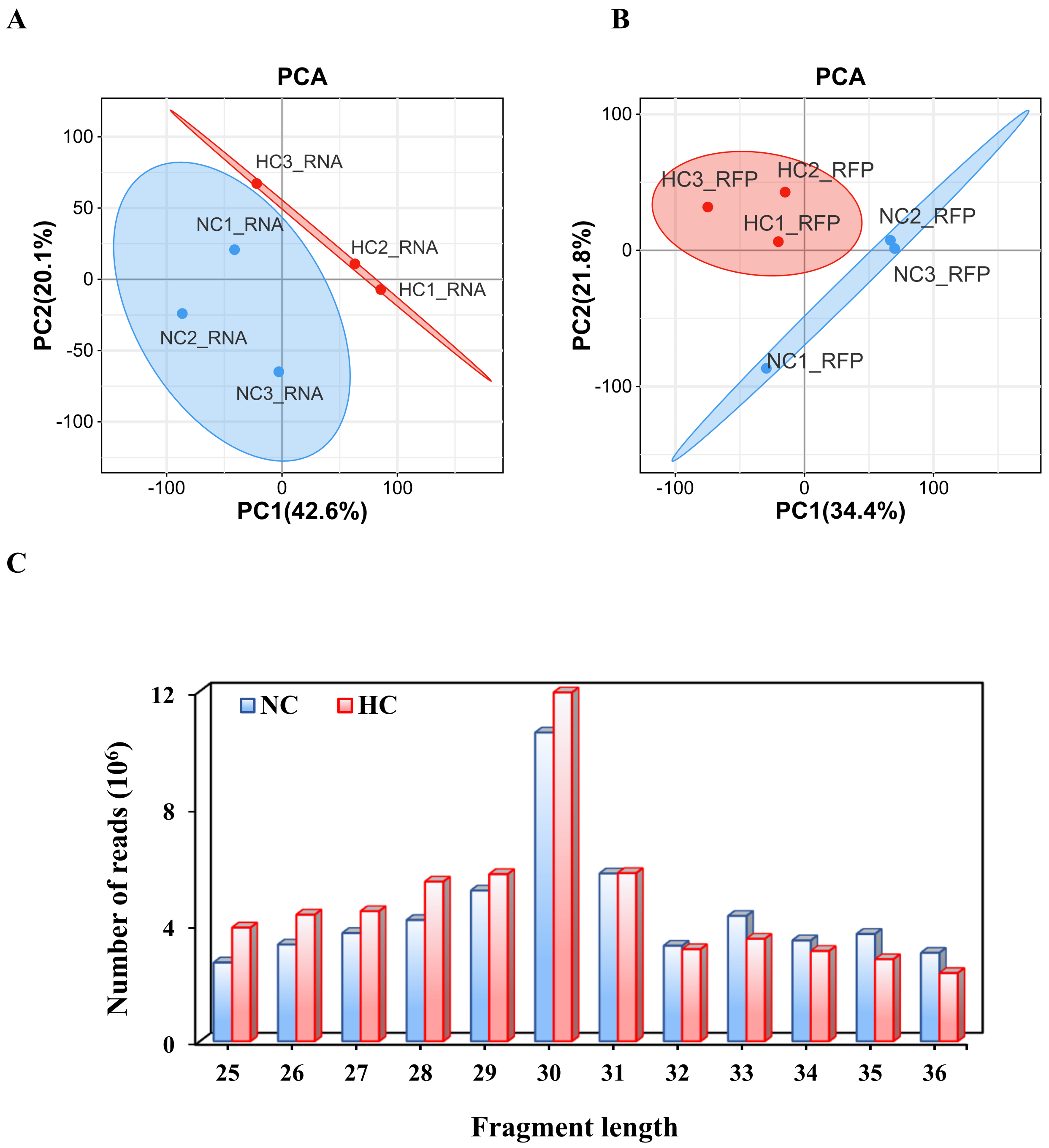

Figure S1. the quality control RNA-seq and Ribo-seq of longissimus dorsi muscle in NC group and HC group.

(A-B) Principal component analysis (PCA) on the RNA-seq (A) and Ribo-seq (B). (C) The percentage of RFPs located in CDS, 5'UTR, and 3'UTR. NC, normal calcium diet; HC, high calcium diet.

Figure S2

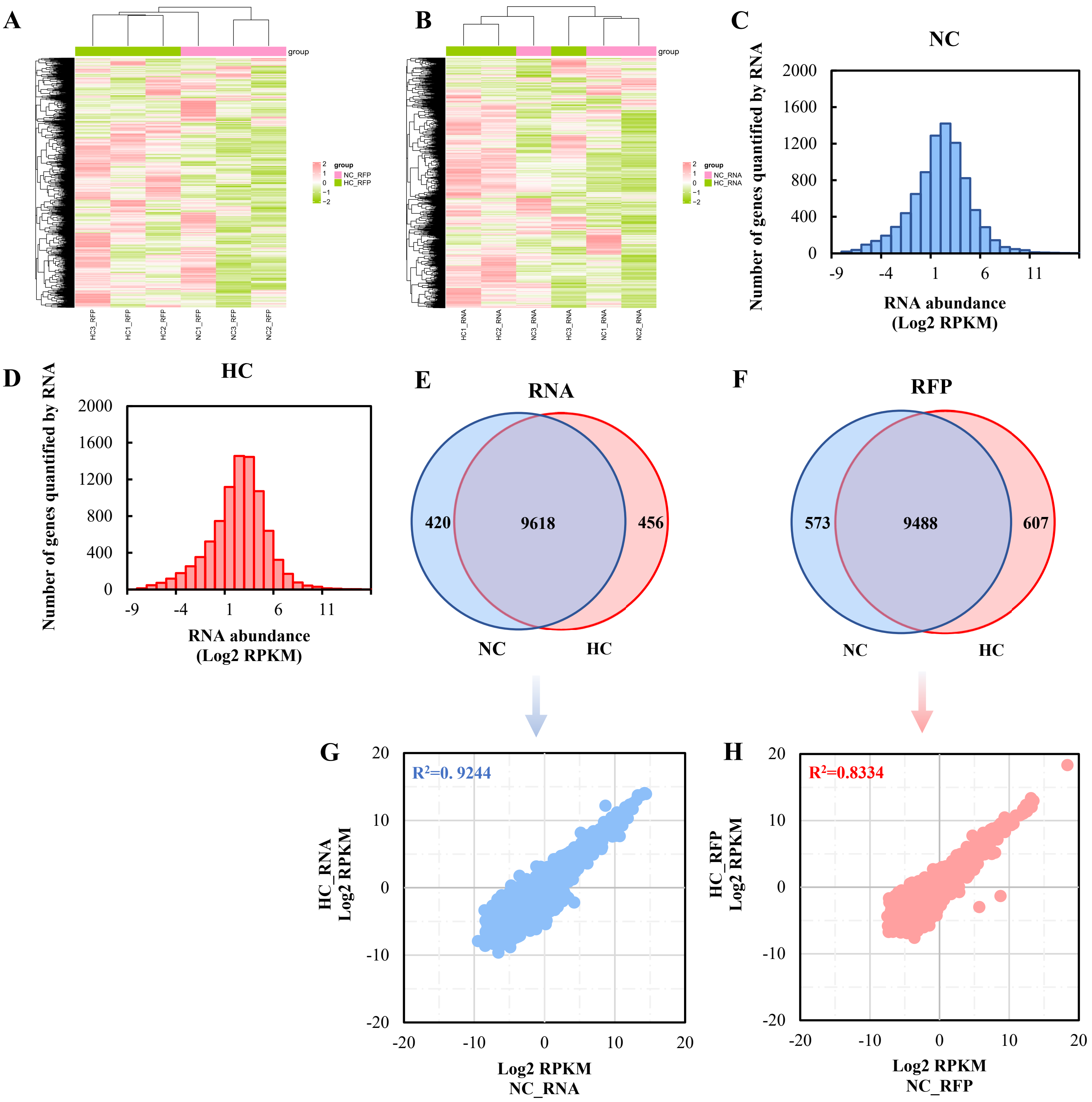

Figure S2. Correlation analysis and the distrbution on RNA and RFP of NC and HC groups. (A-B) Heatmap of the RNA-seq and Ribo-seq in NC and HC groups. (C-D) Distribution of mRNA abundance in NC (C) and HC (D) groups. (E) Venn diagrams of genes identified by RNA-seq of NC and HC groups. (F) Venn diagrams of genes identified by Ribo-seq of NC and HC groups. (G-H) The correlations between the abundances of mRNA and RFP in NC (G) and HC (H) groups.

Figure S3

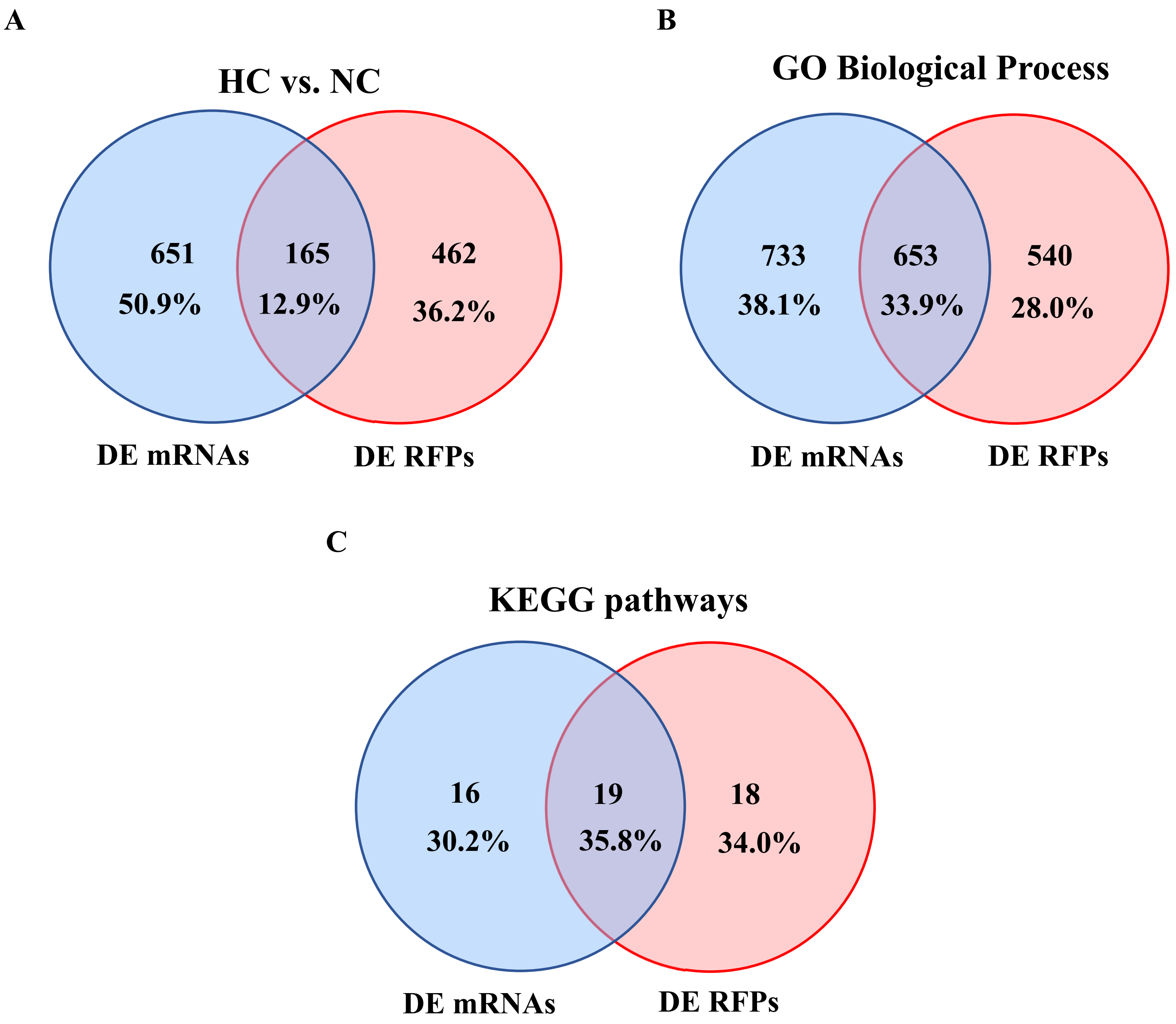

Figure S3. Gene expression was differentially regulated at the transcriptome and translatome levels.

(A) Venn diagram of differentially expressed mRNAs (DE mRNAs) and RFPs (DE RFPs). (B and C) Venn diagrams of significantly enriched GO BPs (B) and KEGG pathways (C) by DE mRNAs and DE RFPs.

Figure S4

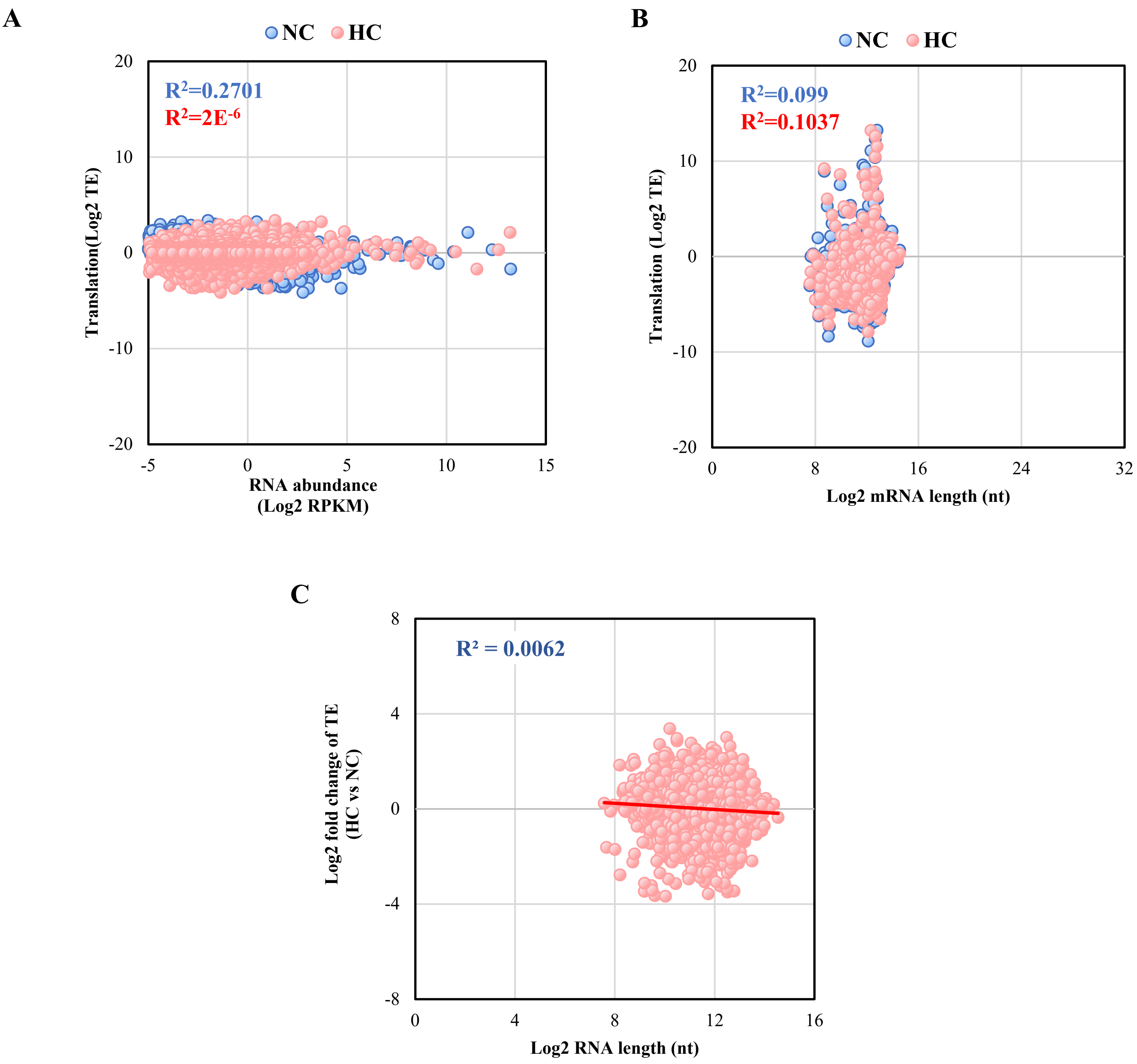

Figure S4. Analysis mRNA translation efficiency between NC and HC groups.

(A) Correlation of mRNA abundance and TE in NC and HC groups, respectively.(B) Correlation of the mRNA max length and TE in NC and HC groups, respectively.(C) Correlation of mRNA length and relative TE (HC vs NC).

Figure S5

A

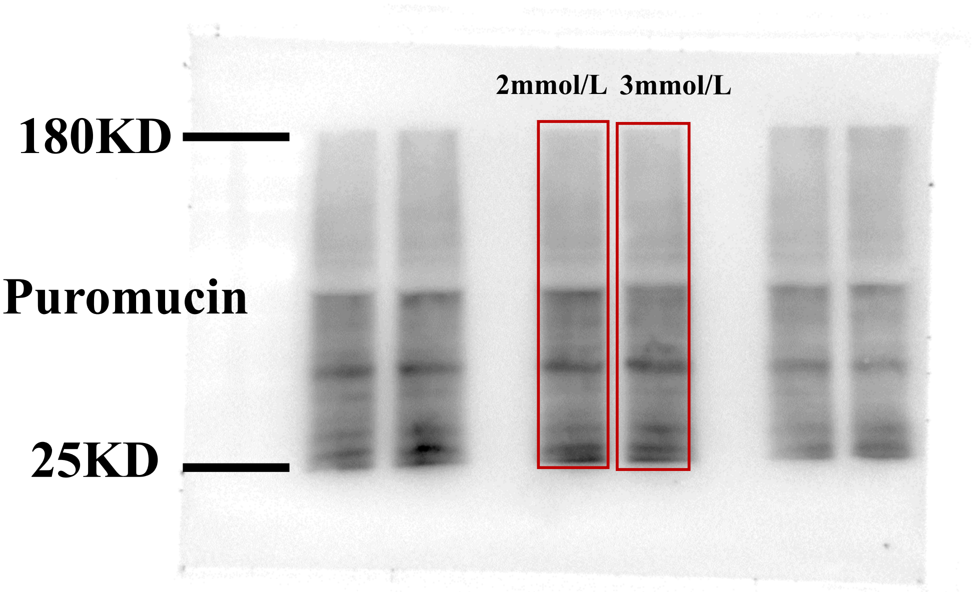

B

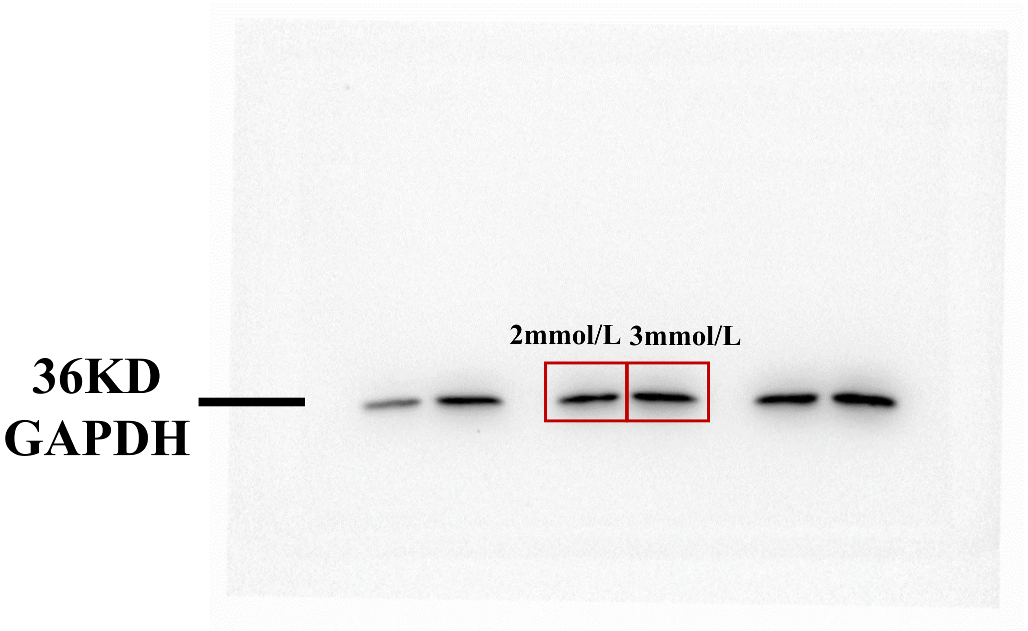

Figure S5. Original gels of Immunoblot of newly synthesized polypeptide labeled by puromycin in C2C12 cells treated with 2mmol/L and 3mmol/L CaCl2.

(A) Original gels image of the Puromycin. (B) Original gels image of the GAPDH.

2 mmol/L, normal calcium level; 3 mmol/L, calcium supplementation.

Figure S6

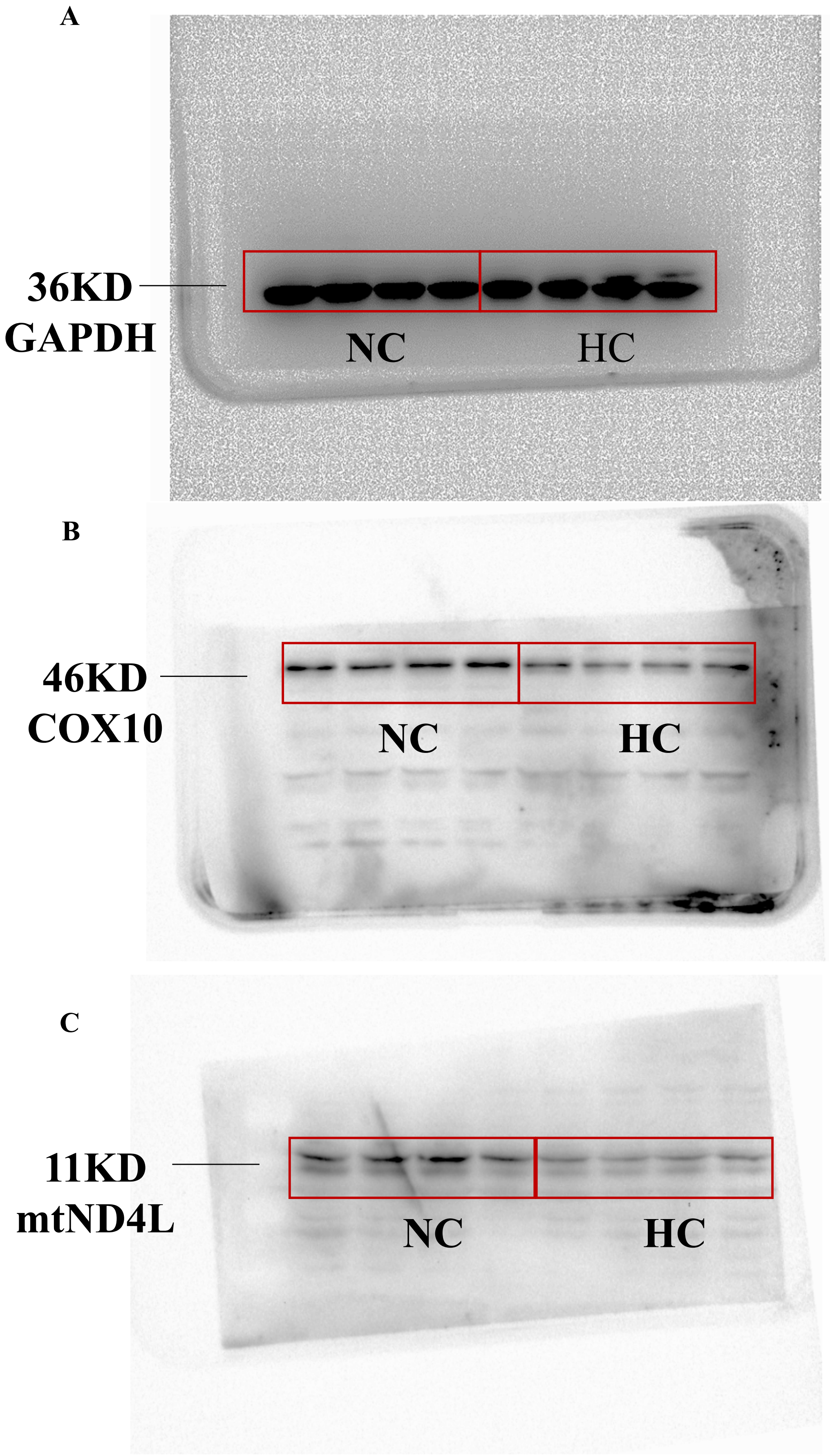

Figure S6. Original gels of WB. (A) Original gels image of the GAPDH.(B) Original gels image of the COX10.(C) Original gels image of the mtND4L. NC, normal calcium diet; HC, high calcium diet.
